# Chemical tuning of photoswitchable azobenzenes: a photopharmacological case study using nicotinic transmission

**DOI:** 10.3762/bjoc.15.274

**Published:** 2019-11-21

**Authors:** Lorenzo Sansalone, Jun Zhao, Matthew T Richers, Graham C R Ellis-Davies

**Affiliations:** 1 Department of Neuroscience, Mount Sinai School of Medicine, New York, NY 10029, USA

**Keywords:** acetylcholine receptors, bidirectional, photoswitchable drug, tetrafluoroazobenzene, visible light

## Abstract

We have developed photochromic probes for the nicotinic acetylcholine receptor that exploit the unique chemical properties of the tetrafluoroazobenzene (4FAB) scaffold. Ultraviolet light switching and rapid thermal relaxation of the metastable *cis* configuration are the main drawbacks associated with standard AB-based switches. We designed our photoprobes to take advantage of the excellent thermodynamic stability of the *cis*-4FAB configuration (thermal half-life > 12 days at 37 °C in physiological buffer) and *cis–trans* photostationary states above 84%. Furthermore, the well-separated n–π* absorption bands of *trans*- and *cis*-4FAB allow facile photoswitching with visible light in two optical channels. A convergent 11-step synthetic approach allowed the installation of a trimethylammonium (TA) head onto the 4FAB scaffold, by means of an alkyl spacer, to afford a free diffusible 4FABTA probe. TAs are known to agonize nicotinic receptors, so 4FABTA was tested on mouse brain slices and enabled reversible receptor activation with cycles of violet and green light. Due to the very long-lived metastable *cis* configuration, 4FAB in vivo use could be of great promise for long term biological studies. Further chemical functionalization of this 4FAB probe with a maleimide functionality allowed clean cross-linking with glutathione. However, attempts to conjugate with a cysteine on a genetically modified nicotinic acetylcholine receptor did not afford the expected light-responsive channel. Our data indicate that the 4FAB photoswitch can be derivatized bifunctionally for genetically-targeted photopharmacology whilst preserving all the favorable photophysical properties of the parent 4FAB scaffold, however, the tetrafluoro motif can significantly perturb pharmacophore–protein interactions. In contrast, we found that the freely diffusible 4FABTA probe could be pre-set with green light into an OFF state that was biologically inert, irradiation with violet light effectively "uncaged" agonist activity, but in a photoreversible manner. Since the neurotransmitter acetylcholine has fully saturated heteroatom valences, our photoswitchable 4FABTA probe could be useful for physiological studies of this neurotransmitter.

## Introduction

Starting in 1937, azobenzenes (ABs) have attracted much attention because they undergo a photoreversible chemical transformation of the thermodynamically favored *trans* configuration into the corresponding *cis* configuration [1]. The reverse reaction can be initiated with a different wavelength. In a biological context, this photochromism was exploited first by Erlanger and colleagues for enzyme inhibitors in 1968 [2], and ion channels in 1971 [3]. Their ingenious approach was utilized further in collaboration with Lester [4–6], but the power of AB photochemistry for biochemistry and cell physiology was then largely forgotten until 2000, when it was revived by Woolley and co-workers in a seminal paper [7]. Since that time, AB photochromes have been exploited by many research groups to control a wide variety of biological process photoreversibly [8–9]. Furthermore, chemists have paid attention to spectral tuning of the AB chromophore [10–11].

Recently, an improved set of fluorine-substituted AB photochromes was developed by Hecht and co-workers [12–13]. ABs with two or four fluorines and electron-withdrawing groups at the *para* substituents all maintained the superb chemical properties of the parent tetrafluoro-(4F)AB chromophore [12]. Mild electron donation effectively destroys the beautiful separation of absorption bands of the n–π* transitions, and with it the excellent high isomeric content of both photostationary states (PSSs). For example, with *para*-diamide substituents the PSS can be either 85% *trans* and 69% *cis* (nitrogen attached to ring [12]) or 96% *trans* and 92% *cis* (carbonyl attached to ring [13]).

**Scheme 1:**
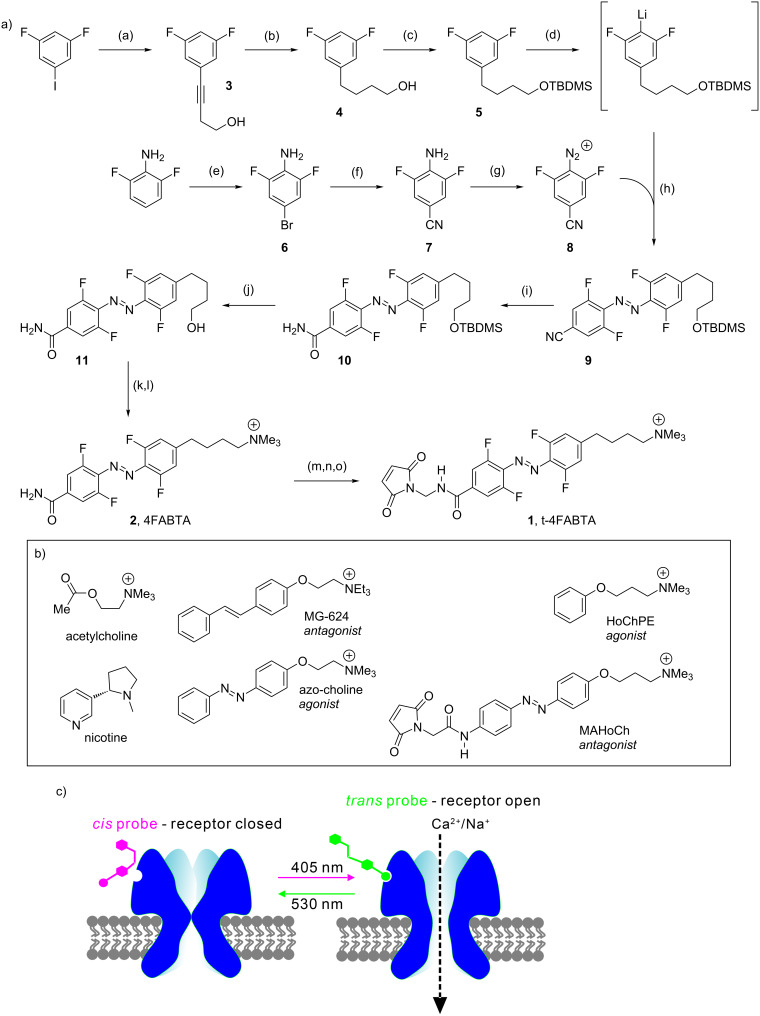
Synthesis of 4FABTA. a) Reagents and conditions: (a) 3-Butynol, PdCl_2_(PPh_3_)_2_, CuI, THF, rt, 93%; (b) H_2_, PtO_2_, EtOH, rt, 71%; (c) TBDMS-Cl, imidazole, DCM, rt, 96%; (d) *n*-BuLi, THF, −78 °C to −50 °C; (e) NBS, ACN, rt, 89%; (f) CuCN, NMP, 202 °C, 70%; (g) NOBF_4_, EtOAC, −10 °C, 54%; (h) THF, −78 °C, 17%; (i) Et_2_NOH, Cu(OAc)_2_, MeOH, rt, 49%; (j) TBAF, THF, rt, 71%; (k) PPh_3_, CBr_4_, THF, rt, 64%; (l) N(CH_3_)_3_, THF, rt, 53% (m) CH_2_O, K_2_CO_2_, H_2_O, 50 °C, 95%; (n) SOCl_2_, THF, −10 °C; (o) maleimide, DIPEA, THF, rt. Counter anion not shown for clarity. b) Structures of nicotinic acetylcholine receptor ligands. c) Illustration of how *cis* and *trans* agonists interact with ligand-gated ion channels.

Since many modern neurobiological applications of AB-based photoprobes require asymmetric substitution of the photochrome, we decided to explore the effects of changing one *para* substituent of 4FAB so as to allow the attachment of different substituents from our first 4FAB photoprobe [14]. Specifically, we were attracted by the application [15] of a regular AB-based photoswitch called "MAHoCh" [16]. The MAHoCh probe was originally designed [16] to activate nicotinic acetylcholine receptors in vivo. However, it was found that the trimethylammonium (TA) head group blocked acetylcholine binding when the photoprobe was cross-linked to the channel near the neurotransmitter binding site, via a (still) mysterious mechanism [15–16]. Normally, TA-based drugs can activate nicotinic acetylcholine receptors, as shown, for example, by Trauner and co-workers [17] (azo-choline, Scheme 1).

Here we explore the effects of addition of the tetrafluoro motif onto such regular AB photoprobes. We have developed a synthetic route that allows the production of asymmetrically substituted 4FAB photoprobes (**1** and **2**, Scheme 1). Importantly, the *cis* and *trans* PSS are both >84%, and the thermal half-life of the *cis* configuration is 12 days in physiological buffer and temperature. The maleimide-substituted compound **1** reacted with the cysteine of glutathione, however, we found that this "4FAB version" of MAHoCh (Scheme 1) did not enable significant perturbation of nicotinic acetylcholine receptor currents used with MAHoCh [16]. However, when 4FABTA analog **2** was tested as a freely-diffusible photoprobe, photopharmacological control of native nicotinic acetylcholine receptors (shown schematically in Scheme 1) with neurons on the medial habenula was possible.

## Results and Discussion

Acetylcholine receptors (AChR) are expressed in many organs, and these receptors fall into two fundamentally different categories which were defined, historically, using pharmacology. The neurotransmitter-gated ion channels are activated by the drug nicotine, and these are called nicotinic (n)AChR. The G-protein-coupled receptors are activated by the drug muscarine, and these are called muscarinic (m)AChR. The structural difference between the native acetylcholine and nicotine are striking, and their diversity reflects the complexity of structure–activity relationships of ligands for nicotinic acetylcholine receptors. For example, the drug homocholine phenyl ether (HoChPE) is an agonist of the nicotinic acetylcholine receptors, but the drug MG-624 is an antagonist (Scheme 1).

**Figure 1:**
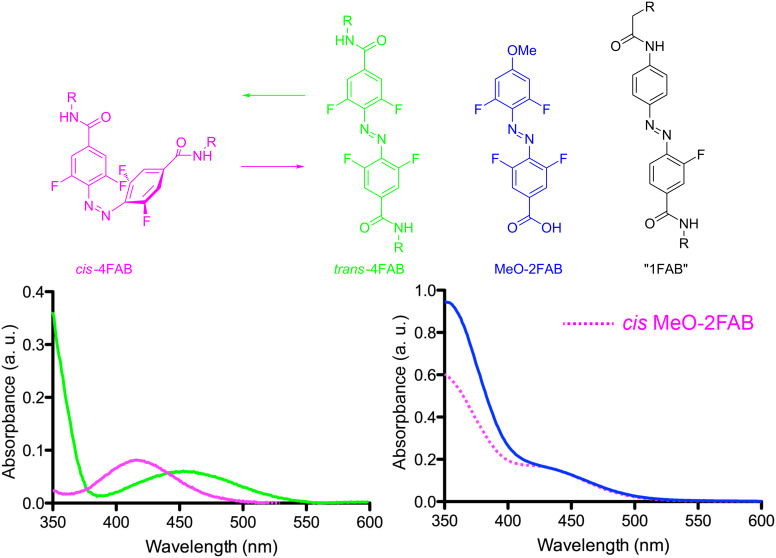
Fluoro-AB derivatives and spectra. Structures of 4FAB-diamides [13] *cis* and *trans* configurations, and the associated absorption spectra of these compounds (left). Note, the analogous difluoro-ABs have similar spectra to 4FABs [13]. Effect of electron donation on the fluoro-AB absorption spectra, the *cis*-MeO-2FAB (pink dots) is the green photostationary state of the photochrome (right). Structure of the photochrome core of a monofluoro-AB (1FAB) [18]. Spectra in HEPES, pH 7.4 at rt.

In our design of photochromes for the nicotinic acetylcholine receptor we were determined to preserve all the excellent chemical properties of the parent 4FAB whilst incorporating this new AB into a "4FAB version" of MAHoCh. Since the latter has an oxygen atom *para* to the azo group we tested the feasibility of adding such a strong electron donor to an analog of a difluoro-(2F)AB Hecht photochrome [13] by making "MeO-2FAB" (Figure 1, see Supporting Information File 1 for synthesis). Since 2FAB and 4FAB photochromes with electron-withdrawing groups at the *para* positions consistently show a separation of the n–π* transitions of the *trans* and *cis* configuration [13], we concluded the presence of one methoxy group in MeO-2FAB effectively destroyed this beautiful separation (a representative example of a 4FAB spectrum is shown in Figure 1). Our data is similar to that recently reported by Gorostiza and co-workers for a monofluorinated AB photochrome [18] (core structure shown in Figure 1 as "1FAB"). This monofluoro-(1)FAB chromophore is much less electron rich than MeO-2FAB, so it is perhaps not surprising that 2FAB cannot tolerate such strong electron-donating group. Hecht and co-workers had shown in their original paper that two amides connected to 2FAB and 4FAB via nitrogen atoms also lost much of the excellent features of the parent 4FAB chromophore [12]. (Note, "1FAB" also has a very short thermal half-life of the *cis* configuration of 10 min at rt [18], compared to 2 years for 4FAB, implying that mild electron donating also destroys the other distinctive property of 4FAB.) Next, we tested if the mildly electron-donating methyl group could be tolerated better by Hecht photochromes [13]. We found that such a derivative did not perturb all the excellent chemical properties of 2FAB (data not shown). Thus, we synthesized a "4FAB version" of MAHoCh (i.e., **1**) as shown in Scheme 1.

The synthesis of photochromes **1** and **2** is outlined in Scheme 1. Sonogashira coupling of difluoroiodobenzene and 3-butynol gave **3**, which was reduced by catalytic hydrogenation to **4** followed by protection with TBDMS to give **5** in 63% yield for three steps. The synthesis of the other half of the photochrome started with bromination of difluorinated aniline to give **6** followed by copper-catalyzed cyanation to **7** in 62% overall yield. Diazonization of **7** with nitrosonium tetrafluoroborate gave **8** (54% yield). The two parts of the chromophore were coupled starting by treatment of **5** with *n*-butyllithium at −78 °C, which was coupled in situ with **8** to AB **9** (17% yield). Copper-catalyzed hydration of **9** gave amide **10**, which was treated with TBAF to alcohol **11** in 42% overall yield. Installation of the cationic head was performed in two steps: first bromination of **11** via Appel reaction, followed by treatment with trimethylamine to give **2** in 35% overall yield. Photoswitch **2** is the first example of a bifunctional, asymmetrically substituted 4FAB chromophore that actually maintains the near-ideal chemical properties of Hecht’s 4FAB chromophores [12–13] (see below). The final synthetic challenge was the installation of a maleimide onto chromophore **2**. The original AB photoswitchable probes which were cross-linked to cysteine mutants had amides with a reverse orientation [19–20] compared to **2**, so a simple one-step route can be used to install the maleimides. It is this functionality that allows coupling (often called "tethering", symbolized as "t" for tetherable in Scheme 1) of the probe with mutant proteins. In our case, we developed a three-step route to install the maleimide using hydroxymethylation with formaldehyde, followed by treatment with thionyl chloride to give a chloromethyl intermediate, which was reacted in situ with maleimide to give **1** in 6% overall yield. Crucially, photochrome **1** also maintained the near-ideal photochemical properties of Hecht’s 4FABs [12–13], specifically the spectral separation of the n–π* transitions of the *trans* and *cis* configurations.

**Figure 2:**
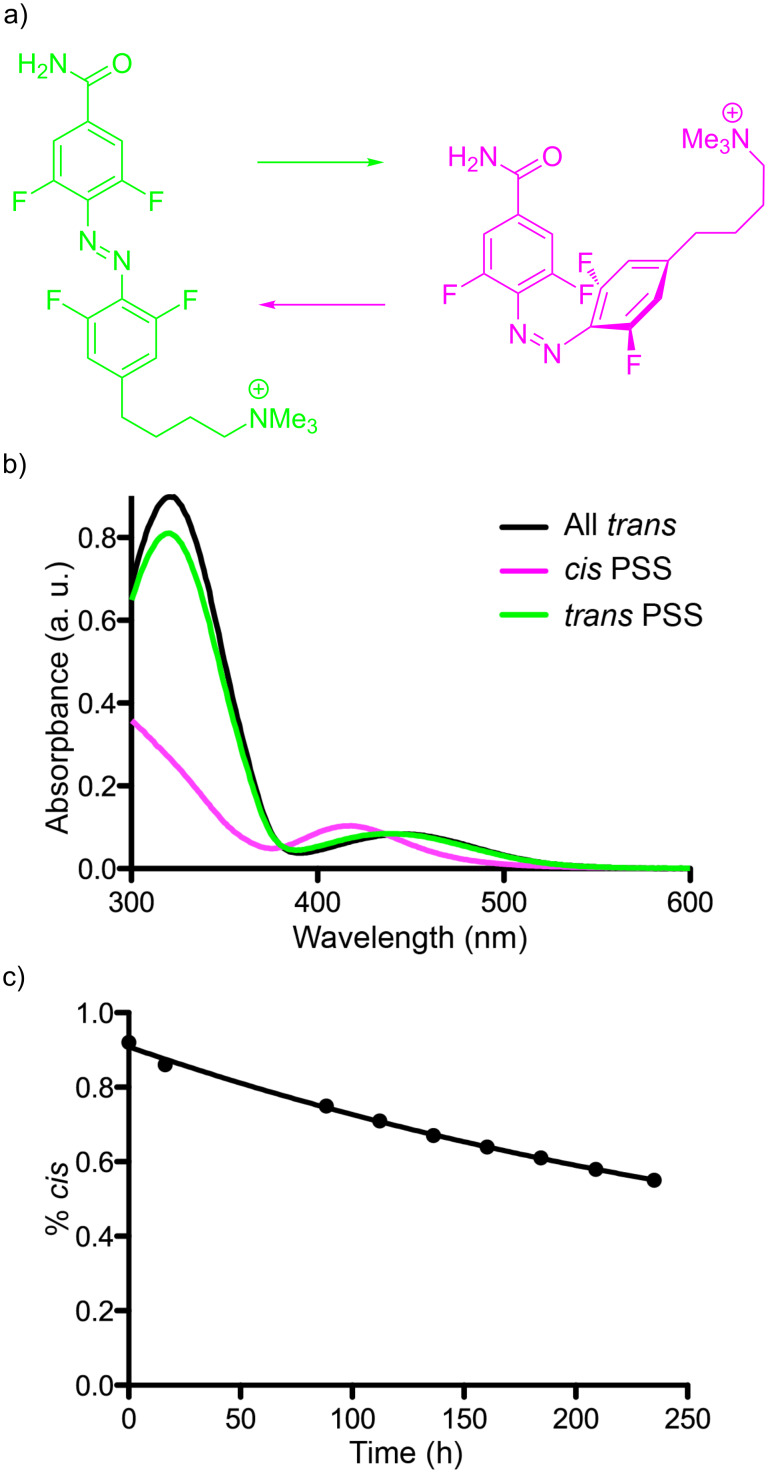
Photochemistry of 4FABTA (**2**), and thermodynamic stability in physiological buffer. a) *Trans*–*cis* photochemical reaction of **2**. b) Absorption spectra of all *trans*-**2** (black), *cis*-**2** PSS (from 532 nm laser), and *trans*-**2** PSS (from 405 nm laser) in HEPES (pH 7.4) at rt. c) Time course of thermal decay of *cis*-**2** PSS in HEPES (pH 7.4) at 37 °C.

Since the maleimide functionality of **1** is quite hydrolytically sensitive at neutral pH, we characterized precursor **2**. This photochrome underwent the expected *trans–cis* isomerization (Figure 2a). The absorption spectrum of the all *trans-****2*** in aqueous solution (HEPES, pH 7.4, no organic co-solvent, Figure 2a) is very similar to the parent 4FAB chromophore (Figure 1). Irradiation with a 532 nm laser gave the *cis* PSS having the expected hypsochromically shifted n–π* band (Figure 2b, full spectra Figure S1, Supporting Information File 1) containing 91% of *cis*-**2** (Figure S2, Supporting Information File 1). Irradiation of this mixture with a 405 nm laser generated the *trans* PSS containing 84% *trans*-**2** (Figure S2, Supporting Information File 1). As already noted, *cis*-4FAB is remarkably thermodynamically stable, some photochromes have a thermal half-life at rt of about 2 years [12]. We used UPLC to measure the thermal half-life of *cis*-**2** under physiological conditions, namely 37 °C and pH 7.4. We found, under these conditions, that *cis*-**2** decayed with a half-life of >12 days (Figure 2c). This datum suggests that even though the electron donor effect of the methylene substituent decreases the thermodynamic stability of the *cis* form compared to that report by Hecht [13], protein conjugates of **1** would be highly stable in vivo over the course of a typical daily mouse behavioral experiment [15]. *cis*-MAHoCh has a significantly shorter thermal half-life of about 75 min at rt [16], a value that would be much lower at physiological temperatures

**Figure 3:**
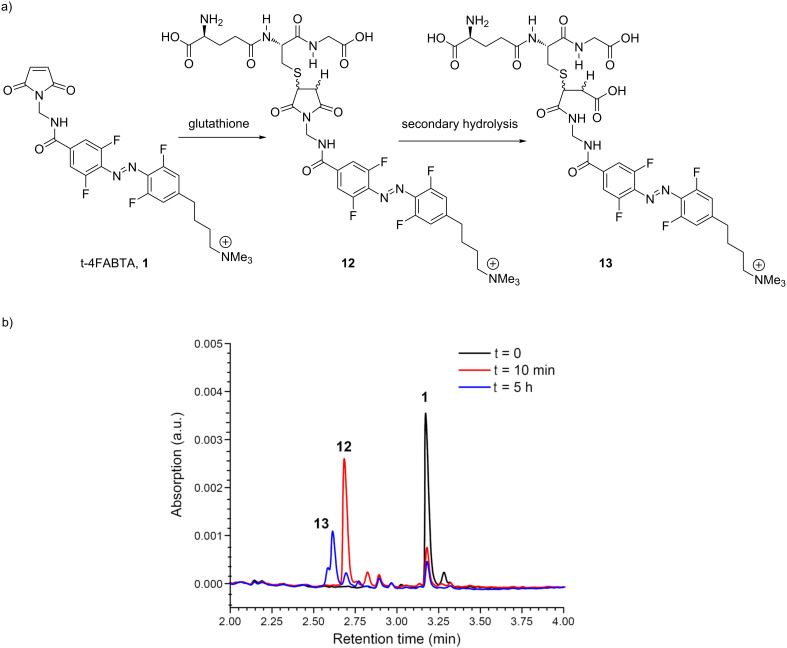
Reaction of t-4FABTA (**1**) with thiols, and thermal stability of initial conjugate. a) Chemical reaction of **1** with glutathione in HEPES (pH 7.4) at 37 °C to coupled conjugate **12**, and hydrolysis to **13**. b) UPLC chromatograms of chemical reactions shown in (a). Elution 0.75 to 1 mL/min, 2–100% acetonitrile in water for 4 min.

Next we tested the chemical reactivity and stability of **1** in physiological buffer. First, we tested the thermal stability of **1** in physiological buffer (HEPES, pH 7.4). We found that in absence of any thiol, the maleimide functionality was reasonably stable, with 80% hydrolysis occurring over 22 h (Figure S3, see also LC–MS in Supporting Information File 1). Reassured by these data, **1** was mixed with a stoichiometric amount of glutathione, at 37 °C rapid reaction gave peak with a shorter retention time on UPLC (Figure 3). LC–MS analysis revealed a molecular ion confirming the addition product **12**. Under the conditions we used for this chemical reaction it was complete within a few minutes. It has been reported that maleimide–peptide conjugates undergo hydrolysis of the succinimide to give a more flexible product of type **13** (Figure 3a) [21]. Thus, we monitored the stability of **12** by UPLC at 37 °C and pH 7.4 and found that it was converted to two new peaks (**13**) in about 5 h (Figure 3b). To our knowledge this is the first report of the stability of a tethered photoswitch [22] under physiological conditions. The broad success [22] of this strategy since 2004 [19] suggests that even though careful in silico modeling is a standard step in photoprobe development [22], this is always performed with unhydrolyzed conjugates. Perhaps for long-term use in vivo an additional modeling step involving hydrolyzed succinimides could be useful.

Our data (Figure 2 and Figure 3) suggested that t-4FABTA (**1**) could be used to replace MAHoCh in the labeling of genetically tagged nicotinic acetylcholine receptors on living cells. Thus, we co-transfected the nicotinic acetylcholine receptors α4 subunit with β2E61C mutant or β2 wild-type (as a control) subunits in HEK293 cells. Cells were treated with **1** for 20 min in the dark, then washed with normal artificial cerebral spinal fluid. First, we compared the basic biophysical properties of cells with mutant and WT β2 subunits, the values were as follows: the resting membrane potential (R_p_) = 29.3 ± 3.9 mV and 28.3 ± 7.6 mV; holding currents at −40 mV were 77.65 ± 30.0 pA, and 76.96 ± 38.9 pA; and the input resistance (R_Input_) = 215.2 ± 36.1 MΩ and 209.1 ± 40.7 MΩ (mutant vs WT, *n* = 5 cells each). These data show treatment with **1** does not change the electrical activity and health of cells.

**Figure 4:**
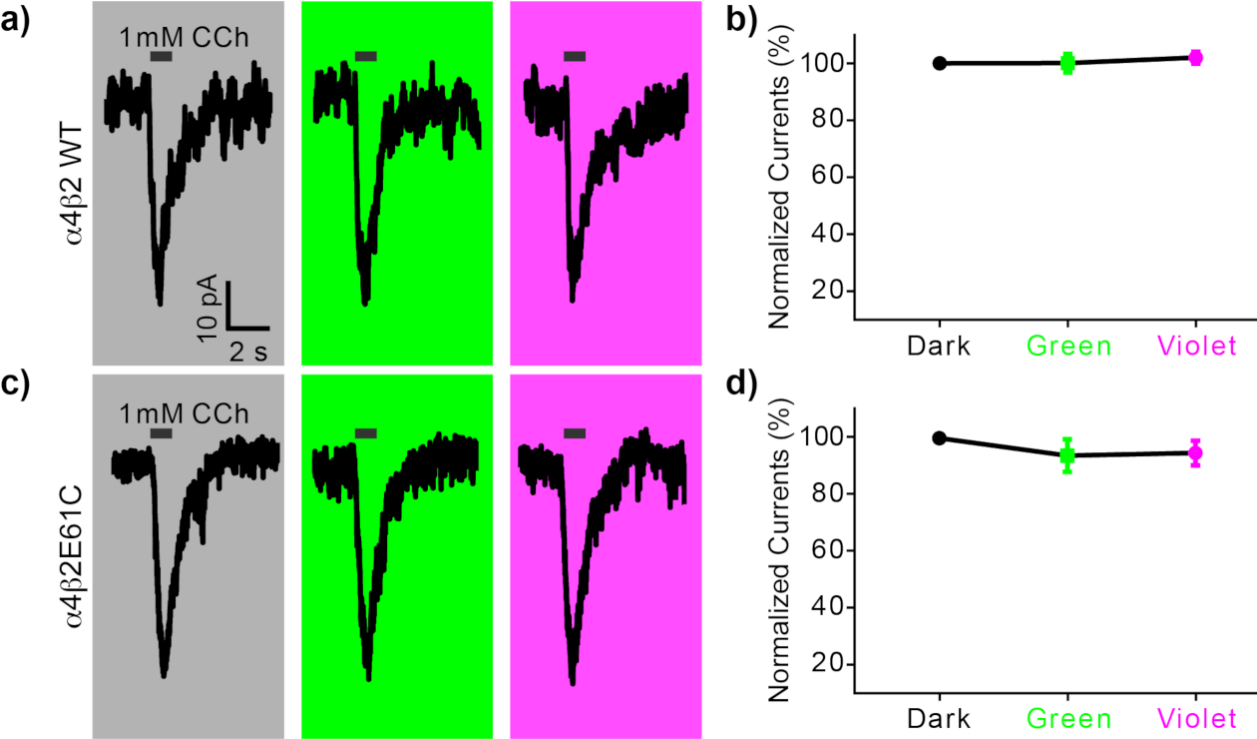
Testing photo-antagonism of **1** with genetically tagged nicotinic acetylcholine receptors. Currents from HEK293 cells were measured using the whole-cell patch-clamp method. Cell were transfected with either wild-type (WT) α4β2 or mutant (E61C) nicotinic acetylcholine receptors [16]. Cells with successful ion channel expression co-expressed YFP visualized using epi-fluorescence microscopy. Cover slips were treated with all *trans*-**1** (0.05 mM in physiological buffer) for 20 min in the dark. The cellular bathing solution was then exchanged for normal buffer before patch clamping. Currents were evoked by local puffing of the agonist carbachol (CCh, 1 mM, black bar). a) Representative currents from WT receptors before illumination (grey), followed by green light (530 nm, 3 mW, 2 min) or violet light (405 nm, 10 mW, 2 min). Irradiation started 100 s prior to puffing and ended 20 s after puffing. b) Summary of four experiments as in (a). Current amplitudes were normalized to the currents in the dark. Data are the mean ± s.e.m, *n* = 4 cells. c) Representative currents recordings from identical experiments to those on WT channels carried out on mutant receptors. d) Summary of the currents from six cells. Current amplitudes were normalized to the currents in the dark. Data are the mean ± s.e.m, *n* = 6 cells.

Next we examined if **1** could serve as a photoswitchable antagonist of nicotinic acetylcholine receptors having α4β2E61C mutant as predicted from previous results [16]. After labeling HEK293 cells as described above, we recorded the currents evoked by puffing the agonist carbachol (CCh) for 1 s under three conditions: no light, or full field irradiation with green or violet light. Both wild-type and mutant channels were tested. Wild-type channels showed substantial inward currents (Figure 4a, grey) which were not perturbed by light (Figure 4a, green and violet, summary Figure 4b). This result was expected as these receptors have no cysteine available for drug conjugation. Surprisingly, similar results were seen with α4β2E61C mutant receptors (Figure 4c,d). We surmise from these data that the mysterious antagonism of *cis*-MAHoCh when conjugated to same mutant receptor is unfortunately perturbed. Since MAHoCh was initially predicted to be a tethered activator in the *trans* configuration, we would conclude the presence of the 4F substituents or the lack of oxygen atom in our version of this photoswitchable probe disturbs crucial drug–protein interactions essential for allosteric inhibition. It is noteworthy to mention that without puffing any CCh agonist, irradiation of green or violet light did not evoke any detectable currents in patch-clamped cells.

**Figure 5:**
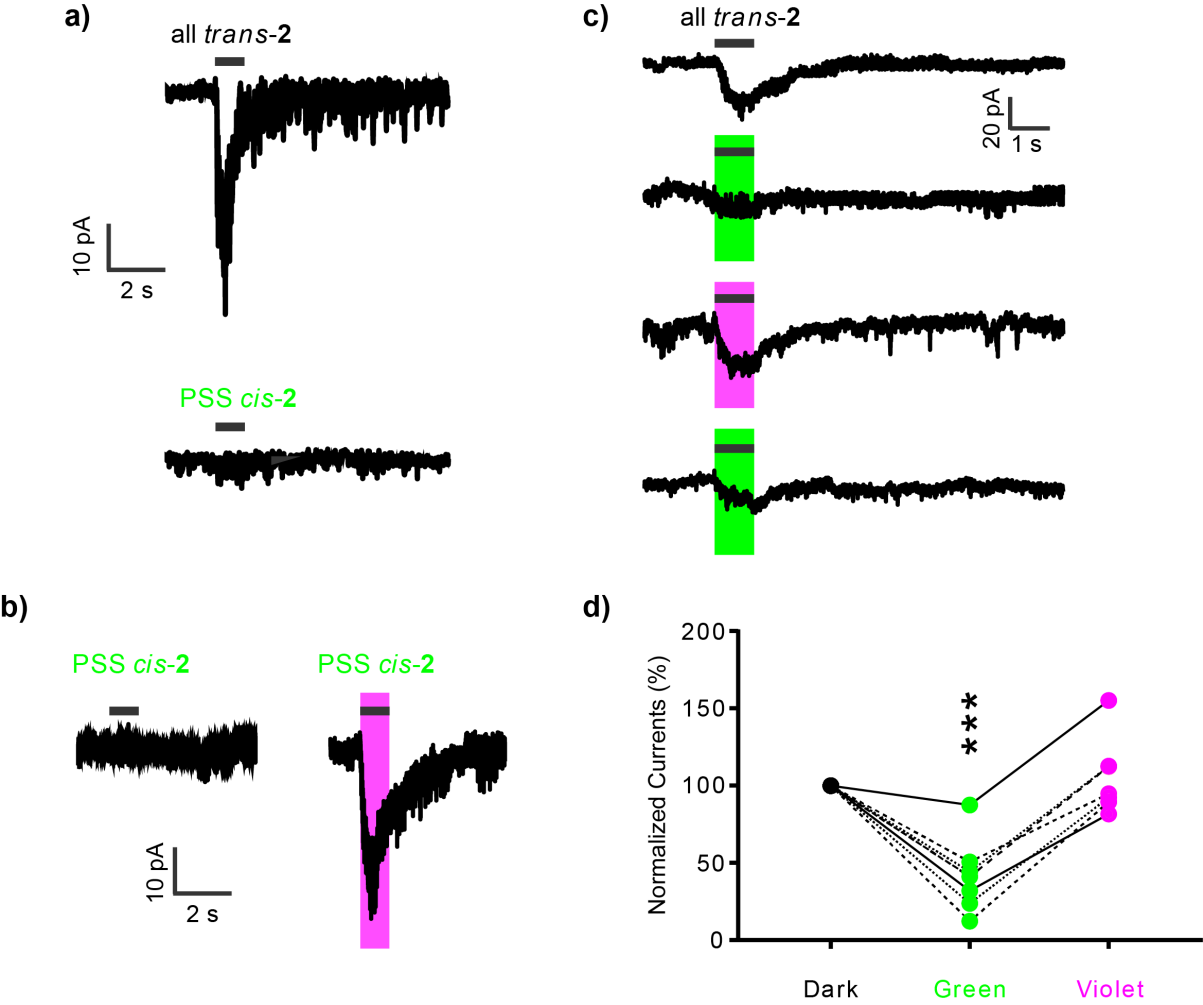
Photopharmacology with 4FABTA (**2**). Currents from neurons in the medial habenula in acutely isolated brain slices were measured using the whole-cell patch-clamp method. Signals from nicotinic acetylcholine receptors were isolated by blocking voltage-dependent Na^+^ channels, muscarinic, GABAergic, and glutamatergic receptors. Photochrome **2** was puffed locally for 1 s (0.2 mM, black bar). LEDs centered at 405 and 530 nm were used for photoswitching. a) Representative responses evoked by all *trans*-**2** and cis-**2** in the green PSS. b) Representative responses evoked by *cis*-**2** in the green PSS without and with violet light (10 mW). c) Sequential responses from the same neuron when all *trans*-**2** was applied without irradiation (top) or with either green or violet light. The interval between sweeps was 3 min. d) Summary of the responses in the first three sweeps (dark-green-violet) from experiments shown in (c). The amplitudes of currents were normalized to the 1st dark response for each cell, *n* = 7 cells, *** indicates *P* < 0.001 in paired t-test.

Finally, we tested the synthetic precursor of our tetherable t-4FABTA probe (i.e., photochrome **2**) as a potential, freely diffusible optical probe of nicotinic acetylcholine receptors in situ. We chose neurons in the medial habenula brain region, as these cells endogenously express a very high density of acetylcholine receptors (mainly α3β4, but also α6, β2, β3 and α4 [23]). First, we puffed all *trans*-**2** (0.2 mM) onto a neuron and recorded excitatory postsynaptic currents (EPSCs) from nicotinic acetylcholine receptors in whole-cell voltage clamp mode. We isolated these currents by including TTX (blocker of voltage-dependent Na^+^ channels), APV and CNQX (antagonists of NMDA and non-NMDA receptors), bicuculline and gabazine (GABAergic antagonists), and atropine (muscarinic acetylcholine receptor antagonist) in the artificial cerebral spinal fluid bathing solution. Thus, puffing all *trans*-**2** for 1 s evoked large EPSCs (ca. 40 pA). In contrast, puffing *cis*-**2** (0.2 mM) in the green PSS (i.e., 91% *cis*) did not evoke any detectable currents (Figure 5a). Next we identified whether *cis*-**2** could be photoswitched quickly to *trans*-**2** to function as an agonist in real time. We puffed *cis*-**2** (0.2 mM) in the green PSS onto a patch-clamped neuron for 1 s, and irradiated the full field of the microscope with violet light. We found that currents were evoked almost as quickly (Figure 5b, right) as those detected from use of all *trans*-**2** (Figure 5a, top). Thus, under these conditions the green PSS functions effectively like a "caged agonist", with violet irradiation rapidly producing an "uncaging-like" biological response. Since the neurotransmitter acetylcholine seems impossible to derivatize with a photochemical protecting group, a biologically inert photo-activatable agonist such **2** could be useful for neurophysiological studies. The long-term thermal stability of the 4FAB core allows a "stock" of the biologically inert *cis* PSS to be made. Since thermal decay is slow at 37 °C, frozen [14] such solutions could be used for many days without any change in pharmacological efficacy.

Our final set of biological experiments with photochrome **2** examined its ability to work as a dynamic, bidirectional photoswitchable drug for nicotinic acetylcholine receptors (Scheme 1c). To test this, we puffed all *trans*-**2** for 1 s onto a patch-clamped medial habenula neuron. As expected this drug configuration evoked an inward current (Figure 5c, upper trace). Irradiation of the application pipette during a subsequent puff with either green or violet light caused rapid photoswitching of **2** (Figure 5c, colored traces). The time between these sweeps was 3 min. Since we used full field illumination through the epi-fluorescence port of the microscope for our experiments, the light not only illuminates an area around the cell, but much of the volume of the puffer pipette reservoir (illustrated in Figure S4, Supporting Information File 1). Figure 5d summarizes 7 independent experiments showing that irradiation of green light significantly decreased the evoked currents (42.8 ± 8.6% of *trans*-evoked currents), while irradiation with violet light reverted evoked currents back to the initial level (106.7 ± 9.1% of initial *trans*-**2**). Taken together, our studies suggest that photochromic drug **2** acts as a fast, reversible photoswitch for modulating the activity of nicotinic acetylcholine receptors on neurons in living brain slices.

## Conclusion

Acetylcholine is one of the two major excitatory neurotransmitters. We have synthesized a photoswitchable tetrafluoroazobenzene (4FAB) agonist of nicotinic acetylcholine receptors that is biologically inert at sub-millimolar levels in the *cis* AB configuration. Photoswitching of the chromophore on neurons with violet light rapidly activates inward currents. We designed our photoprobe to take full advantage of high thermodynamic stability of the *cis* configuration of 4FAB, thus our probe can be pre-set to ON or OFF and used for extended periods without change of function.

## Experimental

### Chemical synthesis

Full experimental synthetic details can be found in Supporting Information File 1.

### Absorption spectroscopy

UV–vis spectra were recorded using a Cary 50 spectrophotometer (Agilent, Santa Clara, CA, USA) at rt in quartz cuvettes with a 1 cm path length in HEPES buffer at pH 7.4.

### UPLC

Chromatography was performed using a Waters Acuity Arc using Cortecs C-18 column (4.6 × 50 mm, 2.7 μm) monitored at 443 nm. Elution was isocratic (Figure S2 and Figure S3, Supporting Information File 1), or used a linear gradient (Figure 3), as specified. Both solvents contained 0.1% TFA.

### Cell culture and electrophysiology

HEK293 cells were split and maintained in 0.1% gelatin-coated coverslips in a 12-well plate with the culture media (DMEM + 10% FBS + 1x Pen Strep). When the confluence reached ≈60%, 0.3 μg NACHO, 0.3 μg α4+eGFP and 0.3 μg β2E61C+eGFP or β2+eGFP WT were co-transfected into cells by Lipofectamine 3000 reagents (Thermo Fisher Scientific, USA) in each well. 2–4 days after transfection, cells simultaneously expressed nACh receptors and eGFP separately due to IRES elements in the vectors were used for photoswitch experiments.

Cells which were labeled in a recording chamber with compound **1** for 20 min, were then washed with external solution. Before and after labeling, labeling solutions were checked by UPLC. The external solution was (in mM): 140 NaCl, 2.8 KCl, 2 CaCl_2_, 2 MgCl_2_, 10 HEPES, 12 glucose (pH 7.3 with NaOH). Compound **1** (0.05 mM) was dissolved in external solution and sonicated for 5 min.

HEK293 cells with green fluorescence were chosen for recording. Patch pipettes were filled with an internal solution containing (in mM): 135 potassium gluconate, 4 MgCl_2_, 10 HEPES, 4 Na_2_-ATP, 0.4 Na_2_-GTP, 10 Na_2_-phosphocreatine, pH 7.35. Good recordings were identified only when resting membrane potential (R_p_) < −20 mV, holding current < 200 pA at −40 mV, and Rs 10–20 MΩ. When recording nACh receptor currents, cells were held at −60 mV. Carbachol (1 mM) was puffed into the cells with a patch pipette for 1 second. For photoswitch, cells were irradiated with green LED (530 nm, 3 mW, 2 min) or violet LED (405 nm, 10 mW, 2 min). Irradiation started 100 s prior to puffing and ended 20 s after puffing.

All animal studies were approved by Mount Sinai IACUC review. C57BL/6J mice (2–3 month old) were anaesthetized with isoflurane and the brain was quickly removed. Coronal brain sections (300 μm) were made in ice-cold cutting solution containing (in mM): 60 NaCl, 2.5 KCl, 1.25 NaH_2_PO_4_, 7 MgCl_2_, 0.5 CaCl_2_, 26 NaHCO_3_, 10 glucose, 100 sucrose, 3 sodium pyruvate, 1.3 sodium ascorbate equilibrated with 95% O_2_/5% CO_2_ (pH 7.3–7.4). The brain slices were then incubated for 15 min at 33 °C in artificial cerebrospinal fluid (ACSF, mM: 125 NaCl, 2.5 KCl, 1.25 NaH_2_PO_4_, 1 MgCl_2_, 2 CaCl_2_, 26 NaHCO_3_, 10 glucose, 3 sodium pyruvate, 1.3 sodium ascorbate; 95% O_2_/5% CO_2_, pH 7.3–7.4) and held at room temperature.

Brain slices were then transferred to the recording chamber of a BX-61 microscope (Olympus, PennValley, PA, USA) and superfused with ACSF at room temperature. Medial habenula neurons were visualized under a 60× objective (Olympus) and infrared differential interference contrast optics. Whole-cell recordings were made with an EPC-10 amplifier using Patchmaster (Heka Instruments, Bellmore, NY, USA) in voltage-clamp mode (*V*_hold_ = −60 mV). Patch pipettes were filled with an internal solution containing (in mM): 135 potassium gluconate, 4 MgCl_2_, 10 HEPES, 4 Na_2_-ATP, 0.4 Na_2_-GTP, 10 Na_2_-phosphocreatine, pH 7.35. The red fluorescent dye Alexa 594 (0.05 mM, ThermoFisher Scientific, Waltham, MA, USA) was added to visualize the morphology of the neurons. Normal ACSF was added with the drug cocktails during photoswitch experiments. The cocktail is 10 μM CNQX, 100 μM APV, 1 μM TTX, 20 μM bicuculline, 20 μM gabazine and 2 μM atropine. For each photoswitch experiment, 0.2 mM 4FABTA (**2**) was locally puffed into a cell concurrently without irradiation or with either 1 s green (530 nm, 3 mW) or 1 s violet light (405 nm, 10 mW).

Data were analyzed in Patchmaster software (Heka). The amplitudes of currents were calculated as the difference of the peak and baseline. All data were present as the mean ± SEM. One-way ANOVA was used to determine whether there are any statistically significant differences between the means of the three groups (i.e., dark, green and violet), and paired t-test was used for post hoc comparisons. *P* < 0.05 indicates statistically significant.

## Supporting Information

File 1 Additional figures, full synthetic details and NMR spectra, LC–MS of compounds **12** and **13** and LC–MS of the hydrolysis of compound **1**.
