# Supplementary material for: Chemical tuning of photoswitchable azobenzenes: a photopharmacological case study using nicotinic transmission
Source: Beilstein J Org Chem. 2019 Nov 21;15:2812–21. doi: 10.3762/bjoc.15.274 (PMC6880823; doi:10.3762/bjoc.15.274)
Supplement: File 1 — Additional figures, full synthetic details and NMR spectra, LC–MS of compounds 12 and 13 and LC–MS of the hydrolysis of compound 1. [file Beilstein_J_Org_Chem-15-2812-s001.pdf]

BEILSTEIN JOURNAL OF ORGANIC CHEMISTRY

Supporting Information

for

Chemical tuning of photoswitchable azobenzenes: a photopharmacological case study using nicotinic transmission

Lorenzo Sansalone, Jun Zhao, Matthew T. Richers and Graham C. R. Ellis-Davies

Beilstein J. Org. Chem. **2019,** *15,* 2812–2821. doi:10.3762/bjoc.15.274

Additional figures, full synthetic details and NMR spectra, LC–MS of compounds 12 and 13 and LC–MS of the hydrolysis of compound 1

License and Terms: This is a supporting information file under the terms of the Creative Commons Attribution License (http://creativecommons.org/licenses/by/4.0). Please note that the reuse, redistribution and reproduction in particular requires that the authors and source are credited.
The license is subject to the *Beilstein Journal of Organic Chemistry* terms and conditions: (https://www.beilstein-journals.org/bjoc)

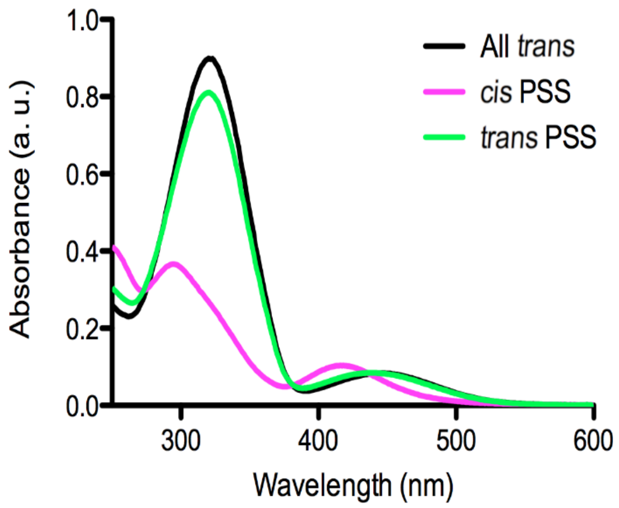

Figure S1: Absorption spectra of 4FABTA (**2**) in HEPES (pH 7.4) at rt.

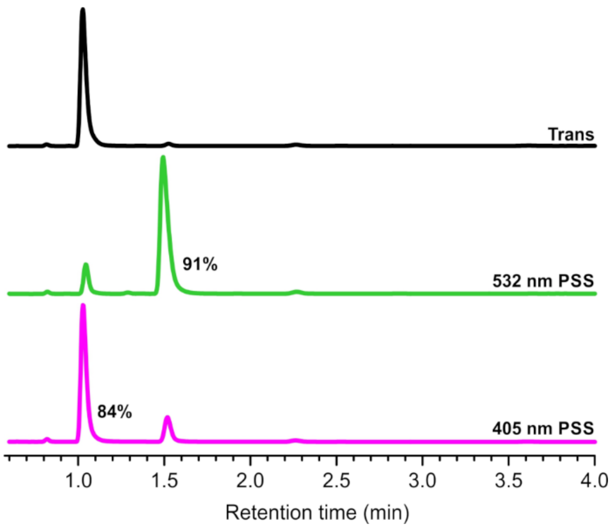

Figure S2: UPLC of PSS of **2**. Isocratic elution at 1.0 mL/min with 30% acetonitrile in water. Laser irradiation (532 or 405 nm) in HEPES (pH 7.4) at rt.

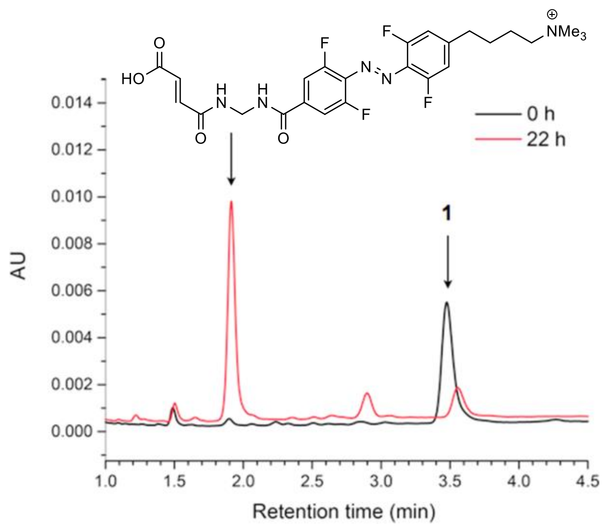

Figure S3: UPLC of maleimide hydrolysis of **1**. Isocratic elution at 1.0 mL/min with 27.5% acetonitrile in water.

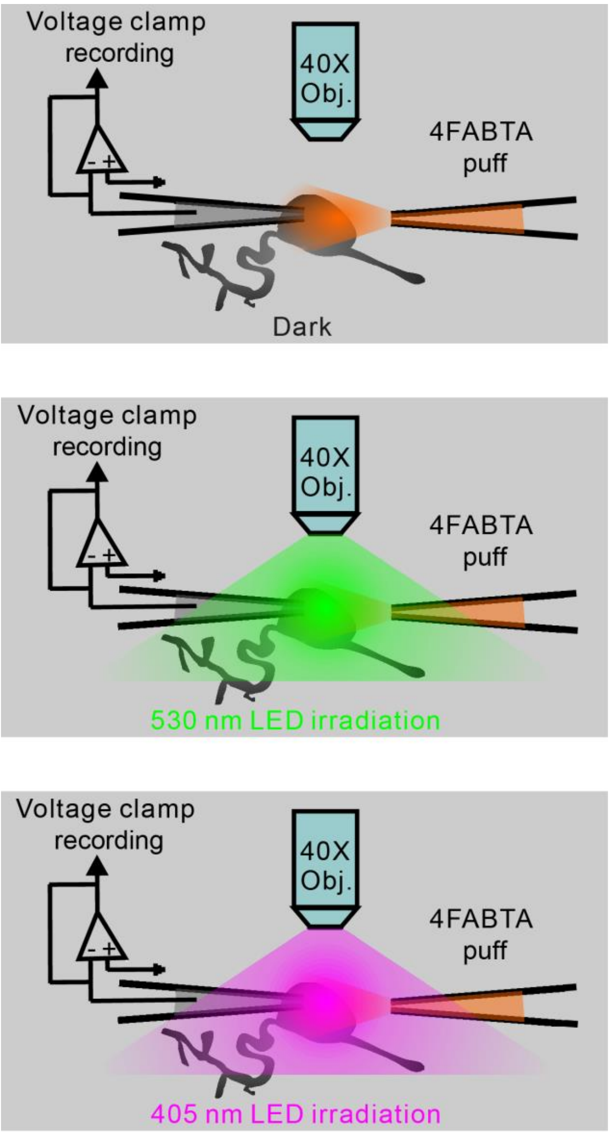

Figure S4: Setup used for experiments in Figure 5. Top – probe was puffed onto a patch-clamped cell without irradiation (Figure 5a). Middle/bottom – probe was puffed onto a patch-clamped cell with concomitant illumination with green of violet light (Figure 5b,c). The field of view of the microscope is about 500 microns, and light completely illuminates this area. Cells are only 10–20 microns in diameter, and thus much of the proximal puffer pipette is also illuminated causing photoswitching of probe inside the pipette “reservoir”.

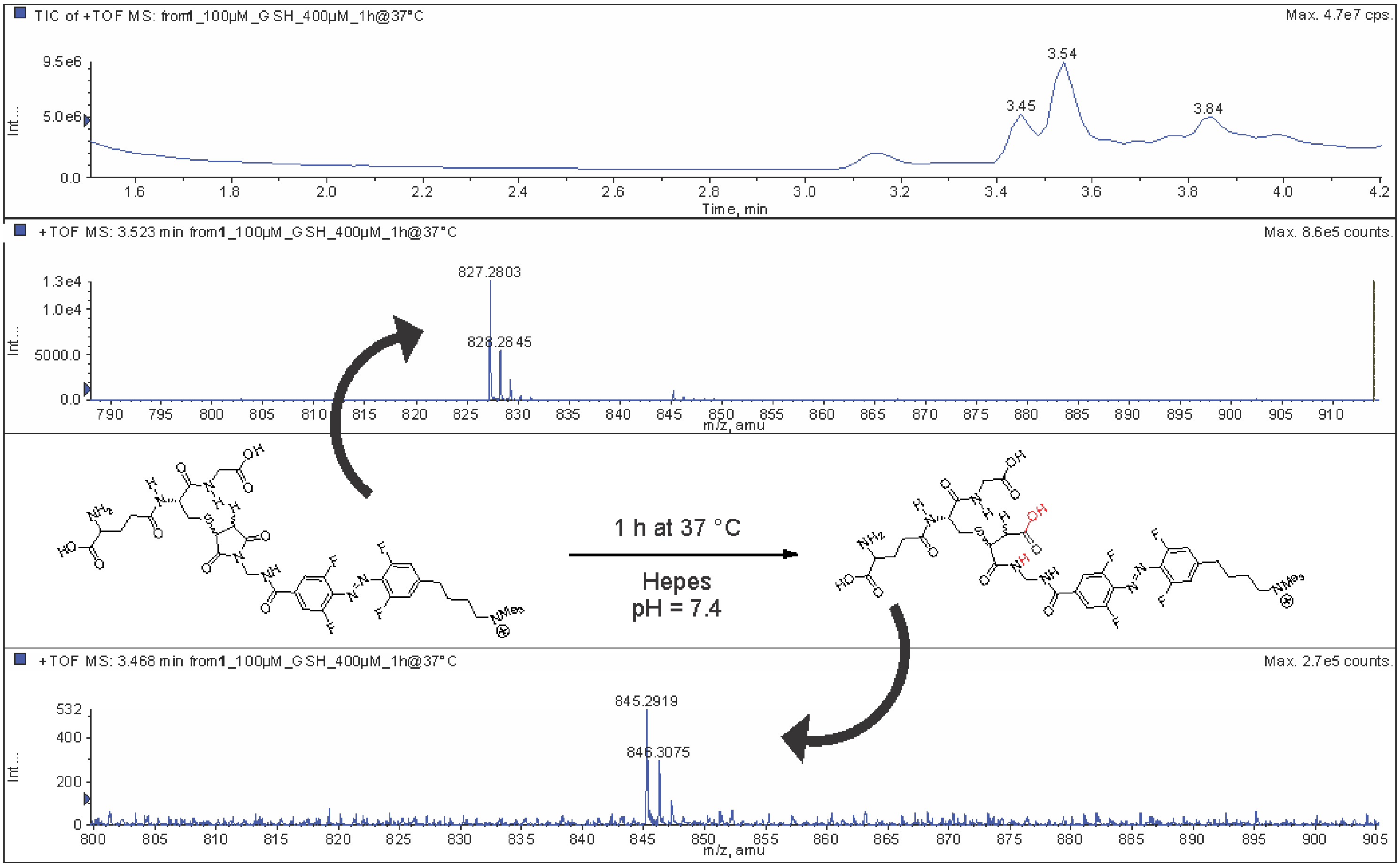

LC–MS for hydrolysis of glutathione-**1** conjugate.

Hydrolyzed 1 in Hepes: HRMS

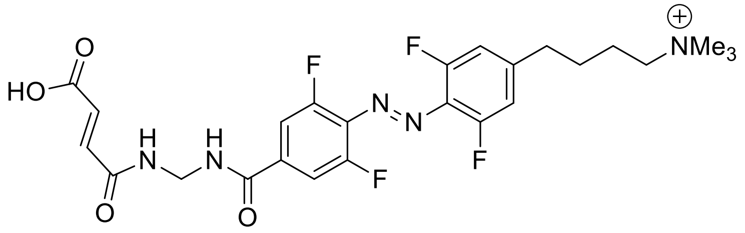

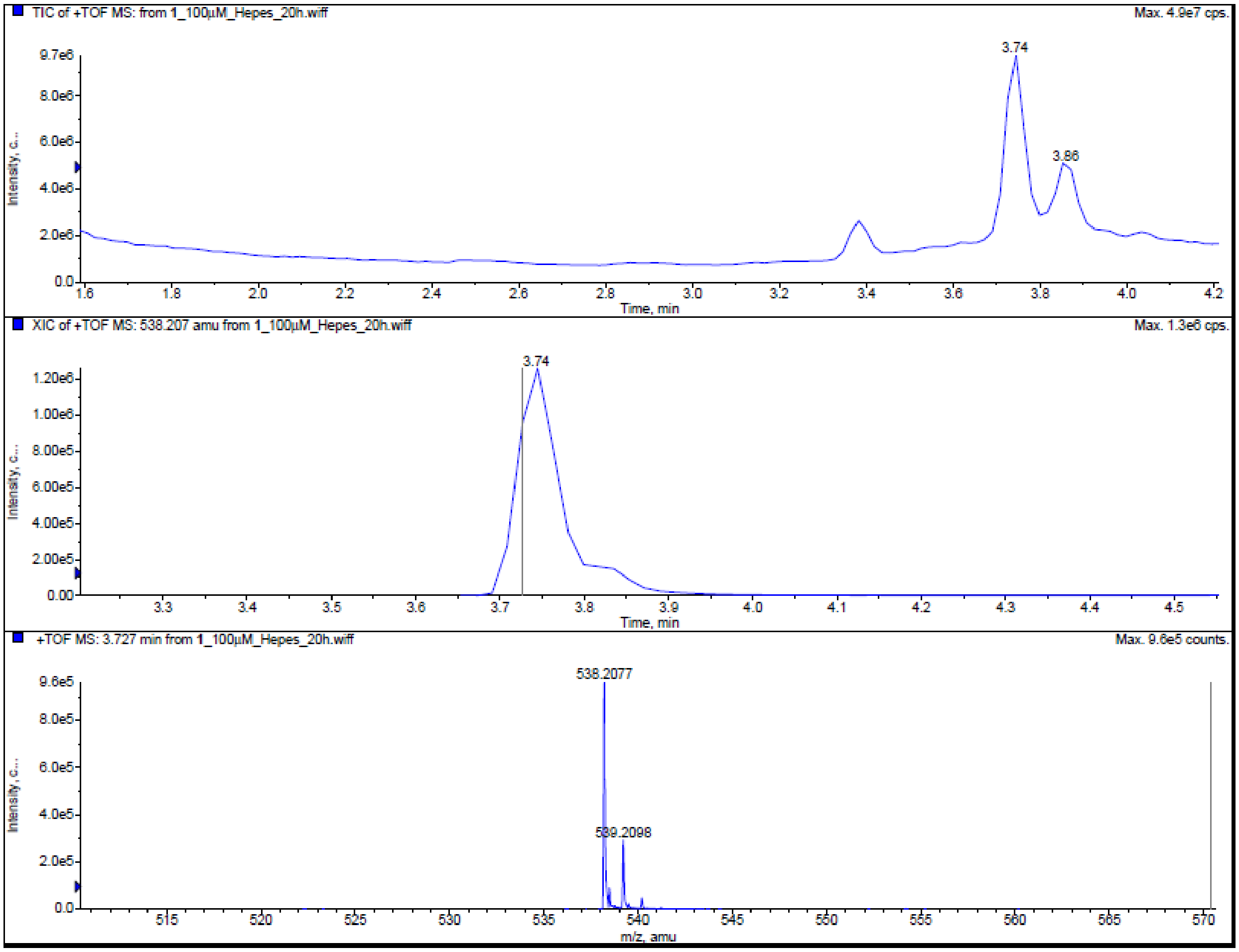

LC–MS for hydrolysis of **1**.

Synthetic methods.

All chemicals were purchased from commercial sources and used as received. Reactions were monitored by thin-layer chromatography (TLC) on Merck KGaA glass silica gel plates (60 F254) and were visualized with UV light or ninhydrin staining followed by heating. Flash chromatography was performed using Agela Technologies industrial grade silica (200–300 mesh, 40–60 μm). Analytical HPLC was performed on a Waters Aquity Arc UPLC system. Proton nuclear magnetic resonance (^{1}H NMR) spectra were recorded on an Oxford 300 MHz NMR spectrometer and the chemical shifts are reported in ppm using the solvent peak as the internal standard (7.26 ppm for $CDCl_3$, 3.31 ppm for CD_3OD, 2.50 ppm for DMSO-d_6, 4.79 ppm for D_2O). Peaks are reported as: s = singlet, br s = broad singlet, d = doublet, t = triplet, q = quartet, dd = doublet of doublets, td = triplet of doublets, m = multiplet. Proton-decoupled carbon nuclear magnetic resonance (^{13}C NMR) spectra were recorded on an Oxford 300 MHz NMR spectrometer and the chemical shifts are reported in ppm using the solvent peak as the internal standard (77.16 ppm for $CDCl_3$, 49.00 ppm for CD_3OD, 39.52 ppm for DMSO-d_6). High resolution mass spectral data for small molecules were obtained on an Agilent G1969A ToF LC–MS.

Synthetic Scheme S1

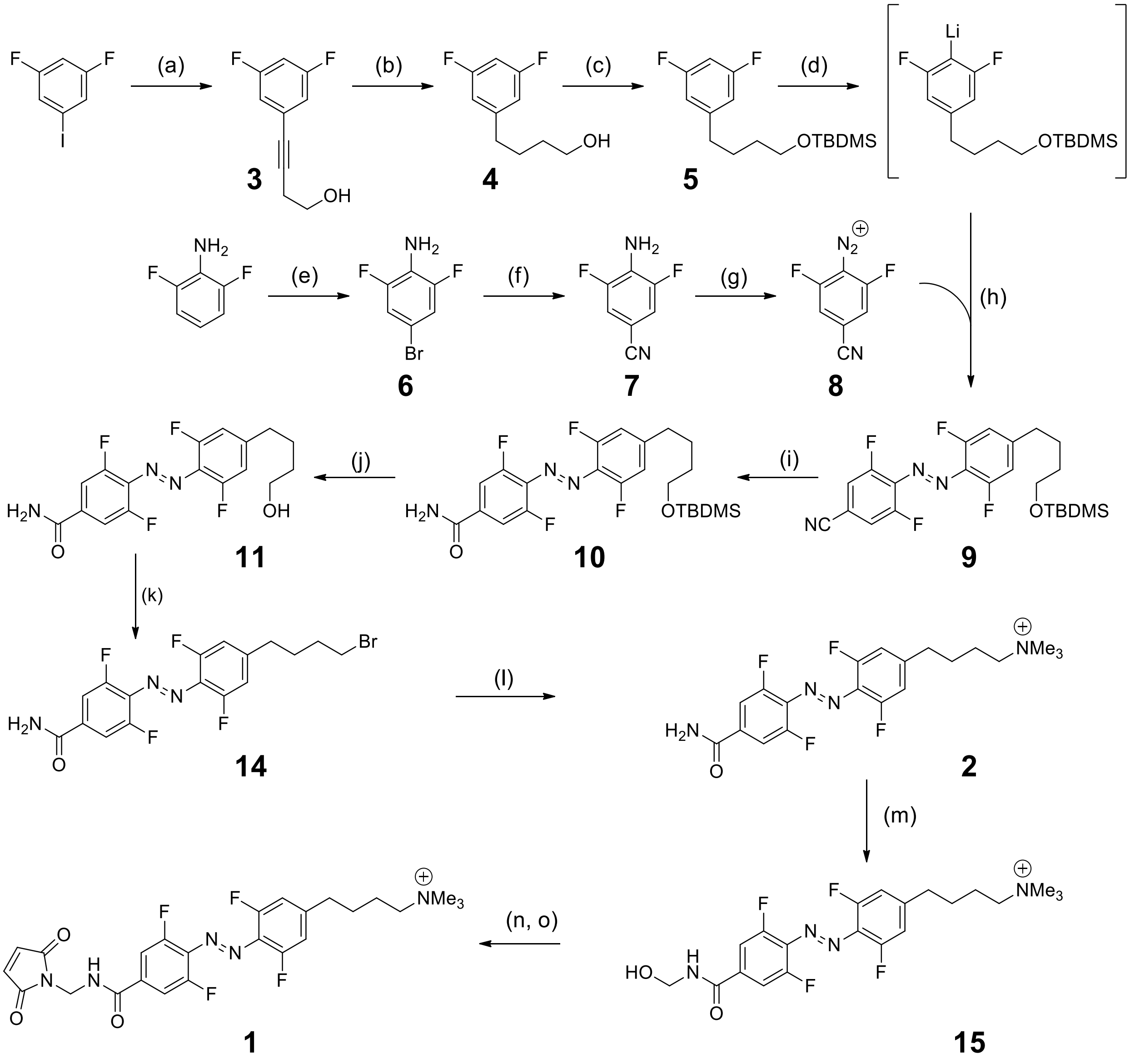

Synthesis of 1. (a) 3-Butynol, $PdCl_2(PPh_3)_2$, CuI, THF, rt, 93%; (b) H_2, PtO_2, EtOH, rt, 71%; (c) TBDMS-Cl, Imidazole, DCM, rt, 96%; (d) *n*-BuLi, THF, −78 °C to −50 °C; (e) NBS, ACN, rt, 89%; (f) CuCN, NMP, 202 °C, 70%; (g) $NOBF_4$, EtOAC, −10 °C, 54%; (h) THF, −78 °C, 17%; (i) Et_2NOH, $Cu(OAc)_2$, MeOH, rt, 49%; (j) TBAF, THF, rt, 76%; (k) PPh_3, CBr_4, THF, rt, 64%; (l) $N(CH_3)_3$, THF, rt, 53% (m) CH_2O, K_2CO_3, H_2O, 50 °C, 95%; (n) $SOCl_2$, THF, −10 °C then (o) Maleimide, DIPEA, THF, rt, 6%;

4-(3,5-Difluorophenyl)-3-butyn-1-ol (3)

$PdCl_2(PPh_3)_2$ (730 mg, 1.04 mmol) was added to a solution of 3,5-difluoroiodobenzene (5 g, 20.83 mmol) in a mixture of dry THF (300 mL) and TEA (36 mL) under Ar atmosphere. After 5 minutes of stirring, CuI (198 mg, 1.04 mmol) was added followed by 3-butynol (1.89 mL, 24.99 mmol). The reaction mixture was stirred at rt for 24 h under Ar atmosphere. The resulting suspension was filtered and the solvent was evaporated at reduced pressure. The reside was purified by column chromatography [SiO_2, 100% DCM] to afford **3** (3.54 g, 93%) as an oily residue. HRMS: m/z calcd for $C_{10}H_9F_2O^+$: 183.0616; found: 183.0616 [M + H]$^+$; ^{1}H NMR (300 MHz, CDCl3): δ = 7.01 – 6.85 (m, 2H), 6.76 (tt, *J* = 9.0, 2.4 Hz, 1H), 3.82 (t, *J* = 6.3 Hz, 2H), 2.68 (t, *J* = 6.3 Hz, 2H), 1.77 (s, 1H); ^{13}C NMR (75 MHz, $CDCl_3$) δ = 162.68 (dd, *J* = 248.2, 13.4 Hz), 126.17 (t, *J* = 11.8 Hz), 115.29 – 114.03 (m), 104.14 (t, *J* = 25.4 Hz), 89.12, 80.22 (t, *J* = 3.9 Hz), 60.87, 23.60.

4-(3,5-Difluorophenyl)-1-butanol (4)

Platinum (IV) oxide (332 mg, 1.46 mmol) was suspended in 90 mL of EtOH and the mixture underwent 5 cycles of vacuum/H_2 to favor adsorption of the gas onto the catalyst. **3** (3.54 g, 19.43 mmol) was added and the reaction mixture was stirred at rt under H_2 atmosphere. After 2 h, the solvent was evaporated at reduced pressure. The resulting residue was dissolved in 50 mL of DCM, filtered through a celite pad and the solvent was evaporated at reduced pressure. The residue was purified by column chromatography [SiO_2, 100% DCM] to afford **4** (2.58 g, 71%) as a light amber oil. HRMS: m/z calcd for $C_{10}H_{13}F_2O^+$: 187.0929; found: 187.0930 [M + H]$^+$; ^{1}H NMR (300 MHz, $CDCl_3$): δ = 6.82 – 6.49 (m, 3H), 3.66 (t, *J* = 6.3 Hz, 2H), 2.63 (t, *J* = 7.4 Hz, 2H), 1.76 – 1.53 (m, 4H), 1.49 (s, 1H); ^{13}C NMR (75 MHz, $CDCl_3$) δ = 163.01 (dd), 146.37 (t, *J* = 8.9 Hz), 112.19 – 109.97 (m), 101.19 (t, *J* = 25.3 Hz), 62.34, 35.41 (t, *J* = 1.9 Hz), 32.03 , 27.06.

***tert*-Butyl(4-(3,5-difluorophenyl)butoxy)dimethylsilane (5)**

Imidazole (2.56 g, 37.59 mmol) was added into a solution of **4** (2.33 g, 12.53 mmol) in 90 mL of DCM and the mixture was stirred at rt. After 5 minutes, TBDMS-Cl (2.27 g, 15.04 mmol) was added and the reaction was stirred at rt for 1 h. The resulting suspension was filtered and the filtrate was washed with $NaHCO_3$ aq. sat. (4 × 40 mL), DI H_2O (1 × 50 mL), dried over Na_2SO_4 and evaporated at reduced pressure to afford **5** (3.62 g, 96%) as a clear oil which was used for the next step without further purification. HRMS: m/z calcd for $C_{16}H_{27}F_2OSi^+$: 301.1794; found: 301.1785 [M + H]$^+$; ^{1}H NMR (300 MHz, $CDCl_3$): δ = 6.77 – 6.51 (m, 3H), 3.62 (t, *J* = 6.3 Hz, 2H), 2.61 (t, *J* = 7.5 Hz, 2H), 1.77 – 1.42 (m, 4H), 0.89 (s, 9H), 0.04 (s, 6H); ^{13}C NMR (75 MHz, $CDCl_3$): δ = 163.12 (dd, *J* = 247.4, 13.0 Hz), 146.70 (t, *J* = 8.9 Hz), 112.55 – 109.16 (m), 101.24 (t, *J* = 25.4 Hz), 62.92 , 35.57 (t, *J* = 2.0 Hz), 32.30 , 27.25 , 26.10 , 18.49 , -5.16.

4-Bromo-2,6-difluoroaniline (6)

Synthesized according to a reported procedure[1]

NBS (6.97 g, 38.73 mmol) was added into a solution of 2,6-difluoroaniline (5 g, 38.73 mmol) in 75 mL of dry ACN under Ar atmosphere. The reaction mixture was stirred at rt. After 24 h, the solvent was evaporated at reduced pressure. The residue was dissolved in DCM and filtered to remove succinimide. The organic phase was washed with $NaHCO_3$ aq. sat. (2 × 50 mL), dried over Na_2SO_4 and evaporated at reduced pressure. The resulting residue was purified by column chromatography [SiO_2, hexane/EtOAc (9:1, v/v)] to afford **6** (7.17 g, 89%) as a white solid. HRMS: m/z calcd for $C_6H_5BrF_2N^+$: 207.9568; found: 207.9570 [M + H]$^+$; ^{1}H NMR (300 MHz, $CDCl_3$): δ = 7.06 – 6.93 (m, 2H), 3.52 (s, 2H).

4-Cyano-2,6-difluoroaniline (7)

Synthesized according to a reported procedure[2]

CuCN (1.29 g, 14.42 mmol) was added into a solution of **6** (2 g, 9.61 mmol) in 6 mL of NMP. The mixture was refluxed for 1.5 h. Upon cooling to rt, 50 mL of NH_4OH aq. sat. were added and the mixture was extracted with toluene (3 × 20 mL). The pooled organic layers were washed with NaCl aq. sat. (2 × 30 mL), dried over Na_2SO_4 and evaporated at reduced pressure. The resulting brown residue was purified by column chromatography [SiO_2, hexane/DCM (1:1, v/v)] to afford **7** (1.04 g, 70%) as a white solid. HRMS: m/z calcd for $C_6H_5BrF_2N^+$: 155.0146; found: 155.0414 $[M + H]^+$; ^1H NMR (300 MHz, $CDCl_3$): δ = 7.21 – 7.07 (m, 2H), 4.27 (s, 2H).

4-Cyano-2,6-difluorobenzenediazonium tetrafluoroborate (8)

7 (2.03 g, 13.17 mmol) was dissolved in 20 mL of EtOAc and cooled to −10 °C with a salt/ice bath. $NOBF_4$ (1.54 g, 13.17 mmol) was added in small portions and the reaction mixture was stirred at this temperature under Ar. After 1 h, the resulting suspension was filtered over a glass filter and the collected solid was washed with Et_2O (2 × 40 mL) and dried under vacuum to afford **8** (1.8 g, 54%) as a white powder. The solid was stored packed under Ar. ^1H NMR (600 MHz, CD_3CN): δ = 8.18 – 7.83 (m, 2H).

(*E*)-4-((4-(4-((*tert*-Butyldimethylsilyl)oxy)butyl)-2,6-difluorophenyl)diazenyl)-3,5-difluorobenzonitrile (9)

A solution of *n*-Buli in hexanes (4.5 mL, 7.2 mmol, 1.6 M) was added dropwise into a solution of **5** (2.13 g, 7.1 mmol) dissolved in 10 mL of dry THF at −78 °C. The reaction mixture was stirred at −50 °C for 30 minutes and cannulated into a solution of **8** (1.79 g, 7.1 mmol) in 10 mL of dry THF at −78 °C. The reaction was stirred to rt and 20 mL of $NaHCO_3$ aq. sat. were added. The mixture was extracted with EtOAc (3 × 20 mL) and the pooled organic layers were washed with NaCl aq. sat. (2 × 20 mL), dried over Na_2SO_4 and evaporated at reduced pressure. The residue was purified by column chromatography [SiO_2, hexane/EtOAc (20:1, v/v)] to afford **9** (544 mg, 17%) as a red viscous liquid. HRMS: m/z calcd for $C_{23}H_{28}F_4N_3OSi^+$: 466.1933; found: 466.1935 $[M + H]^+$; ^1H NMR (300 MHz, $CDCl_3$): δ = 7.24 – 7.12 (m, 2H), 6.79 – 6.61 (m, 2H), 3.61 (t, *J* = 6.1 Hz, 2H), 2.59 (t, *J* =z 7.6 Hz, 2H), 1.78 – 1.40 (m, 4H), 0.88 (s, 9H), 0.03 (s, 6H); ^{13}C NMR (75 MHz, $CDCl_3$): δ =153.35 (t, *J* = 6.0 Hz), 149.95 (dd, *J* = 5.8, 2.8 Hz), 147.88 (t, *J* = 8.6 Hz), 135.86 (t, *J* = 17.3 Hz), 129.59 (t, *J* = 17.1 Hz), 117.20 – 115.86 (m), 112.95 (t, *J* = 10.9 Hz), 112.17 (dd, *J* = 20.0, 2.9 Hz), 62.74 , 35.49 , 32.19 , 26.85 , 26.06 , 18.47 , -5.17.

(*E*)-4-((4-(4-((*tert*-Butyldimethylsilyl)oxy)butyl)-2,6-difluorophenyl)diazenyl)-3,5-difluorobenzamide (10)

Net_2OH (0.32 mL, 3.21 mmol) and $Cu(OAc)_2$ (3.6 mg, 0.02 mmol) were respectively added to a solution of **9** (500 mg, 1.07 mmol) in 10 mL of MeOH. The mixture was stirred at rt. After 20 h, the solvent was evaporated at reduced pressure and the residue was purified by column chromatography [SiO_2, hexane/EtOAc (1:1, v/v)] to afford **10** (253 mg, 49%) as an orange solid. HRMS: m/z calcd for $C_{23}H_{30}F_4N_3O_2Si^+$: 484.2038; found: 484.2041 $[M + H]^+$; ^1H NMR (300 MHz, $CDCl_3$): δ = 7.63 – 7.38 (m, 2H), 7.01 – 6.78 (m, 2H), 6.13 (s, 2H), 3.64 (t, *J* = 6.1 Hz, 2H), 2.69 (t, *J* = 7.6 Hz, 2H), 1.83 – 1.44 (m, 4H), 0.90 (s, 9H), 0.05 (s, 6H); ^{13}C NMR (75 MHz, $CDCl_3$): δ = 166.24 , 157.35 (dd, *J* = 59.6, 4.4 Hz), 153.87 (dd, *J* = 58.1, 4.5 Hz), 149.83 (t, *J* = 9.8 Hz), 135.55 (t, *J* = 8.4 Hz), 134.19 (t, *J* = 10.5 Hz), 129.59 (t, *J* = 9.4 Hz), 112.68 (dd, *J* = 19.9, 3.2 Hz), 112.00 (dd, *J* = 23.1, 2.6 Hz), 62.79 , 35.86 , 32.22 , 26.99 , 26.10 , 18.49 , -5.15.

(*E*)-4-((2,6-Difluoro-4-(4-hydroxybutyl)phenyl)diazenyl)-3,5-difluorobenzamide (11)

A solution of TBAF in THF (0.58 mL, 1 M) was added into a solution of **10** (233 mg, 0.48 mmol) in 8 mL of THF. The reaction was stirred at rt. After 16 h, 40 mL of NaCl aq. sat. were added and the mixture was extracted with EtOAc (3 × 15 mL). The pooled organic layers were dried over Na_2SO_4 and evaporated at reduced pressure. The residue was purified by column chromatography [SiO_2, 100% EtOAc] to afford **11** (135 mg, 76%) as a solid. HRMS: m/z calcd for $C_{17}H_{16}F_4N_3O_2^+$: 370.1174; found: 370.1172 $[M + H]^+$; ^{1}H NMR (300 MHz, DMSO-d_6): δ = 8.26 (s, 1H), 7.88 – 7.70 (m, 3H), 7.34 – 7.14 (m, 2H), 4.41 (t, *J* = 5.2 Hz, 1H), 3.42 (q, *J* = 6.0 Hz, 2H), 2.69 (t, *J* = 7.6 Hz, 2H), 1.77 – 1.32 (m, 4H); ^{13}C NMR (75 MHz, DMSO-d_6): δ = 164.50 , 156.19 (dd, *J* = 54.5, 4.7 Hz), 152.75 (dd, *J* = 52.5, 4.6 Hz), 150.67 (t, *J* = 9.9 Hz), 137.45 (t, *J* = 8.2 Hz), 132.29 (t, *J* = 10.6 Hz), 128.46 (t, *J* = 9.6 Hz), 112.95 (dd, *J* = 19.8, 3.1 Hz), 112.18 (dd, *J* = 22.1, 3.0 Hz), 60.35 , 34.76 , 31.82 , 26.51.

(*E*)-4-((2,6-Difluoro-4-(4-bromobutyl)phenyl)diazenyl)-3,5-difluorobenzamide (14)

Ph_3P (164 mg, 0.626 mmol) and CBr_4 (198 mg, 0.596 mmol) were respectively added to a solution of **11** (110 mg, 0.29 mmol) in 5 mL of dry THF. The reaction was stirred at rt under Ar. After 24 h, additional Ph_3P (164 mg) followed by CBr_4 (198 mg) were added and the reaction was stirred at rt for 24 h. The resulting suspension was filtered and the solvent was evaporated at reduced pressure. The residue was purified by column chromatography [SiO_2, hexane/EtOAc (1:2, v/v)] to afford **12** (82 mg, 64%) as an orange solid. HRMS: m/z calcd for $C_{17}H_{15}BrF_4N_3O^+$: 432.0330; found: 432.0334 $[M + H]^+$; ^{1}H NMR (300 MHz, DMSO-d_6): δ = 8.26 (s, 1H), 7.92 – 7.70 (m, 3H), 7.35 – 7.20 (m, 2H), 3.57 (t, *J* = 6.3 Hz, 2H), 2.73 (t, *J* = 7.2 Hz, 2H), 1.93 – 1.63 (m, 4H); ^{13}C NMR (75 MHz, DMSO-d_6): δ = 164.49 , 156.20 (dd, *J* = 54.9, 4.5 Hz), 153.44 – 151.96 (m), 150.01 (t, *J* = 10.2 Hz), 137.65 – 137.18 (m), 132.27 (t, *J* = 10.4 Hz), 128.56 (t, *J* = 9.5 Hz), 113.03 (dd, *J* = 20.0, 3.0 Hz), 112.19 (dd, *J* = 22.0, 2.9 Hz), 34.68 , 33.86 , 31.52 , 28.41.

(*E*)-4-(4-((4-Carbamoyl-2,6-difluorophenyl)diazenyl)-3,5-difluorophenyl)-*N*,*N*,*N*-trimethylbutanaminium bromide (2)

To a solution of **11** (59 mg, 0.137 mmol) in 3 mL of dry THF was added NMe_3 in EtOH (65 µL, 0.27 mmol, 4.2 M). The reaction was stirred at rt. After 12 h, the resulting suspension was filtered to recover 10 mg of **2** as a solid. The filtrated solvent was added with NMe_3 in EtOH (700 µL, 2.94 mmol, 4.2 M) and stirred at reflux for 18 h. The resulting suspension was filtered to recover additional 26 mg of **2**. The pooled solid was dried under vacuum to afford **2** (36 mg, 53%) as an orange powder. HRMS: m/z calcd for $C_{20}H_{23}F_4N_4O^+$: 411.1803; found: 411.1803 $[M + H]^+$; ^{1}H NMR (300 MHz, DMSO-d_6): δ = 7.86 – 7.75 (m, 2H, Phenyl-H), 7.38 – 7.26 (m, 2H, Phenyl-H), 3.38 – 3.22 (m, 2H, -CH_2-NR_3, overlaps with water signal in DMSO-d6), 3.04 (s, 9H, -$N^+(CH_3)_3$), 2.77 (t, *J* = 6.7 Hz, 2H, Phenyl-CH_2-), 1.68 (m, 4H, -CH_2-CH_2-); ^{13}C NMR (201 MHz, DMSO-d_6): δ = 164.46 , 155.72 – 154.54 (m), 154.29 – 153.34 (m), 149.48 , 137.56 , 132.24 , 128.66 , 113.50 – 112.68 (m), 112.55 – 111.97 (m), 64.91 , 52.18 , 34.06 , 26.51 , 21.35.

(*E*)-4-(4-((2,6-Difluoro-4-((hydroxymethyl)carbamoyl)phenyl)diazenyl)-3,5-difluorophenyl)-*N*,*N*,*N*-trimethylbutanaminium trifluoroacetate (15)

2 (32 mg, 0.065 mmol) was added to 1 mL of a 4% K_2CO_3 in H_2O solution and, while stirring, 1 mL of a 37% CH_2O in H_2O solution was added. The reaction mixture was warmed to 50 °C and stirred at this temperature for 2 h. Upon cooling to rt, the solvent was removed at reduced pressure and the resulting residue was purified by column chromatography [SiO_2, MeOH/TFA (100:0.2, v/v)] to afford **13** (32 mg, 95%) as a red solid. HRMS: m/z calcd for $C_{21}H_{25}F_4N_4O_2^+$: 441.1909; found: 441.1916 $[M + H]^+$; ^{1}H NMR (300 MHz, CD_3OD): δ = 7.85 – 7.56 (m, 2H), 7.28 – 6.98 (m, 2H), 4.85 – 4.73 (m, 2H), 3.98 (s, 1H), 3.42 – 3.35 (m, 2H), 3.13 (s, 9H), 2.82 (t, *J* = 7.3 Hz, 2H), 2.04 – 1.66 (m, 4H).

(*E*)-4-(4-((4-(((2,5-Dioxo-2,5-dihydro-1*H*-pyrrol-1-yl)methyl)carbamoyl)-2,6-difluorophenyl)diazenyl)-3,5-difluorophenyl)-*N*,*N*,*N*-trimethylbutan-1-aminium trifluoroacetate (1)

Thionyl chloride (27 µL, 0.378 mmol) was added dropwise to a solution of **13** (30 mg, 0.054 mmol) in 1 mL of dry THF at −10 °C. The reaction mixture was stirred to rt, under Ar, until complete disappearance of the starting material (checked by UPLC). To the stirring mixture were added, respectively, maleimide (16 mg, 0.162 mmol) and DIPEA (11 µL, 0.065 mmol) dissolved in 1 mL of dry THF. The reaction was stirred overnight. The solvent was evaporated at reduced pressure and the residue was purified by column chromatography [C_{18}-SiO_2, Gradient: ACN/H_2O (10:90, v/v to 20:80, v/v) + 0.5% TFA] to afford **1** (2 mg, 6%) as an orange solid. HRMS: m/z calcd for $C_{25}H_{26}F_4N_5O_3^+$: 520.1967; found: 520.1963 $[M + H]^+$; ^1H NMR (300 MHz, CD_3CN) δ = 7.63 – 7.49 (m, 2H, Phenyl-H), 7.28 – 7.01 (m, 2H, Phenyl-H), 6.81 (s, 2H, Maleimide-H), 5.09 (d, *J* = 5.8 Hz, 2H, R_2N-CH_2-Maleimide), 3.30 – 3.16 (m, 2H, -CH_2-NR_3), 2.99 (s, 9H, -$N^+(CH_3)_3$), 2.77 (t, *J* = 7.5 Hz, 2H, Phenyl-CH_2-), 1.79 – 1.61 (m, 4H, -CH_2-CH_2-).

Synthetic Scheme S2

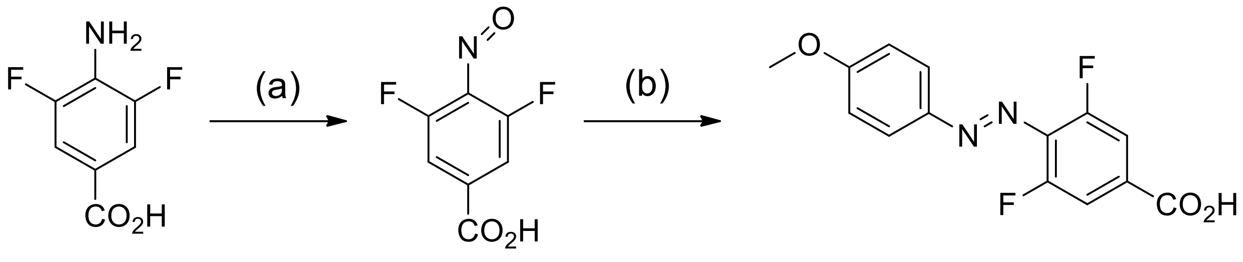

MeO-2FAB

Synthesis of MeO-2FAB. (a) Oxone monopersulfate, H_2O/Acetone/DCM, RT, 60%; (b) AcOH/Toluene/TFA, RT, 18%

(*E*)-3,5-Difluoro-4-((4-methoxyphenyl)diazenyl)benzoic acid (MeO-2FAB)

Oxone monopersulfate (2.13 g, 3.46 mmol) was dissolved in 14 mL of DI H_2O and added to a solution of 4-amino-3,5-difluorobenzoic acid (300 mg, 1.73 mmol) in 6 mL of DCM/acetone (2:1). The mixture was vigorously stirred at rt. After 2 h, 40 mL of DI H_2O were added and the mixture was extracted with EtOAc (3 × 10 mL). The pooled organic layers were dried over Na_2SO_4 and the solvent was evaporated at reduced pressure. The resulting beige solid (nitrosobenzoic acid) was suspended in 12 mL of AcOH/toluene/TFA (6:6:1) and *p*-methoxyaniline (135 mg, 0.64 mmol) was added. The mixture was stirred at rt for 3 days. 60 mL of DI H_2O were added and the mixture was extracted with EtOAc (3 × 35 mL). The pooled organic layers were dried over Na_2SO_4 and the solvent was evaporated at reduced pressure. The residue was purified by column chromatography [SiO_2, DCM/MeOH (90:10, v/v)] to afford **MeO-2FAB** (90 mg, 18%) as a brownish solid. HRMS: m/z calcd for $C_{14}H_{11}F_2N_2O_3^+$: 293.0733; found: 293.0713 $[M + H]^+$; ^1H NMR (300 MHz, CD_3OD): δ = 7.99 – 7.86 (m, 3H), 7.77 – 7.66 (m, 3H), 7.15 – 7.04 (m, 3H), 3.92 (s, 3H). ^{19}F NMR (282 MHz, $CDCl_3$): δ = -120.83 (d, *J* = 8.8 Hz).

Proton NMR of 1 (CD3CN)

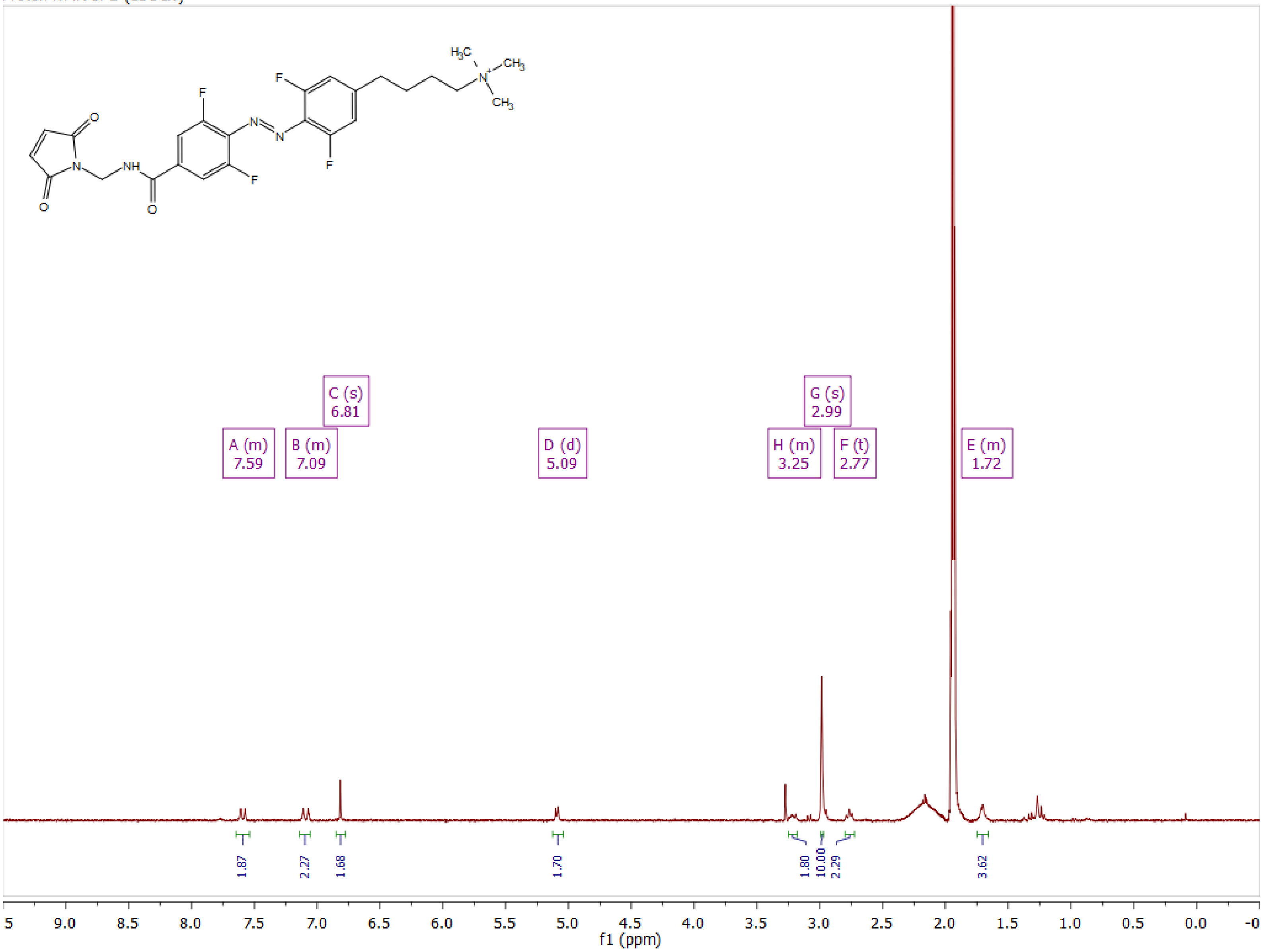

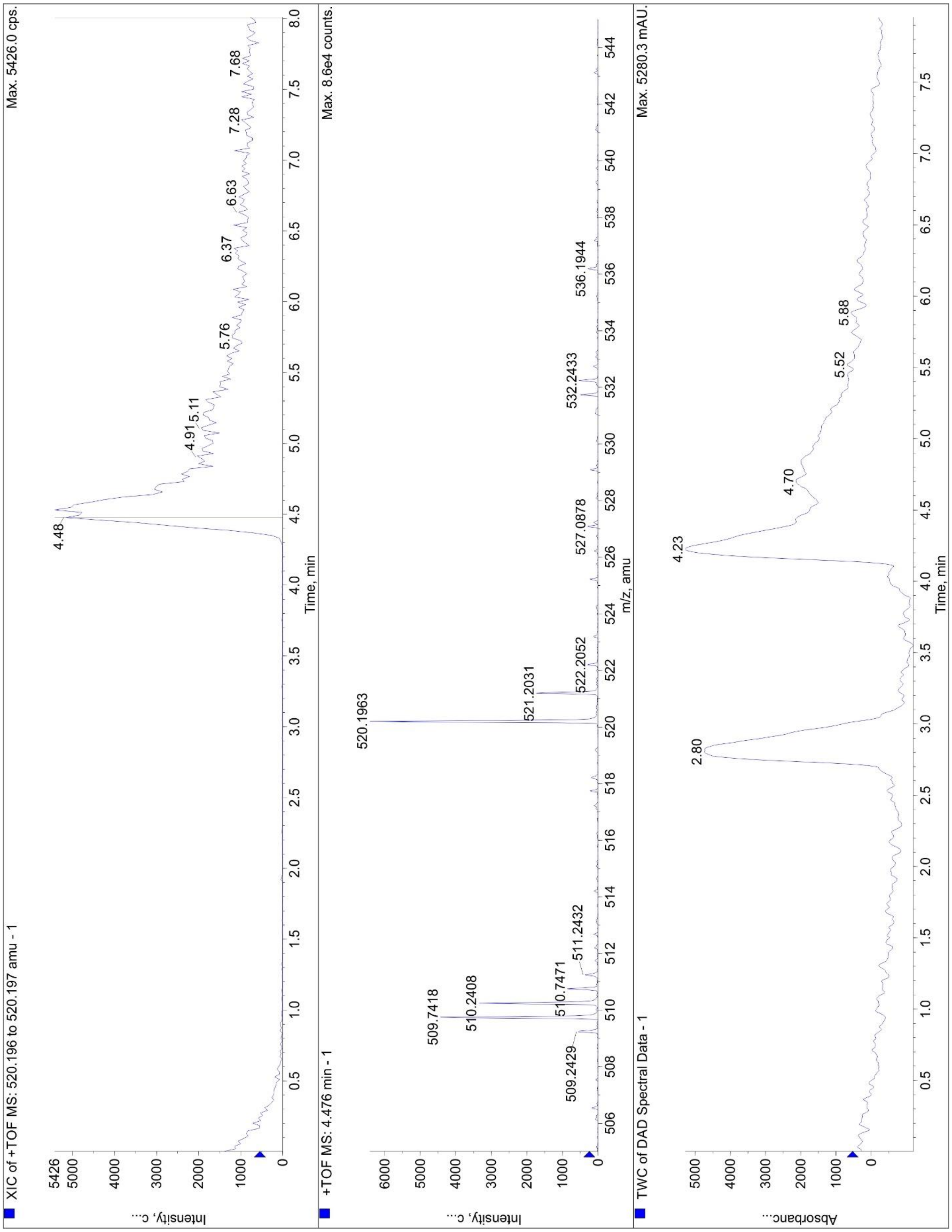
XIC of +TOF MS: 520.196 to 520.197 amu - 1
Max. 5426.0 cps.
Intensity, c...
Time, min
4.48
4.91
5.11
5.76
6.37
6.63
7.28
7.68
+TOF MS: 4.476 min - 1
Max. 8.6e4 counts.
m/z, amu
509.2429
509.7418
510.2408
510.7471
511.2432
520.1963
521.2031
522.2052
527.0878
532.2433
536.1944
TWC of DAD Spectral Data - 1
Max. 5280.3 mAU.
Absorbanc...
2.80
4.23
4.70
5.52
5.88

Proton NMR of 2 (DMSO-d6)

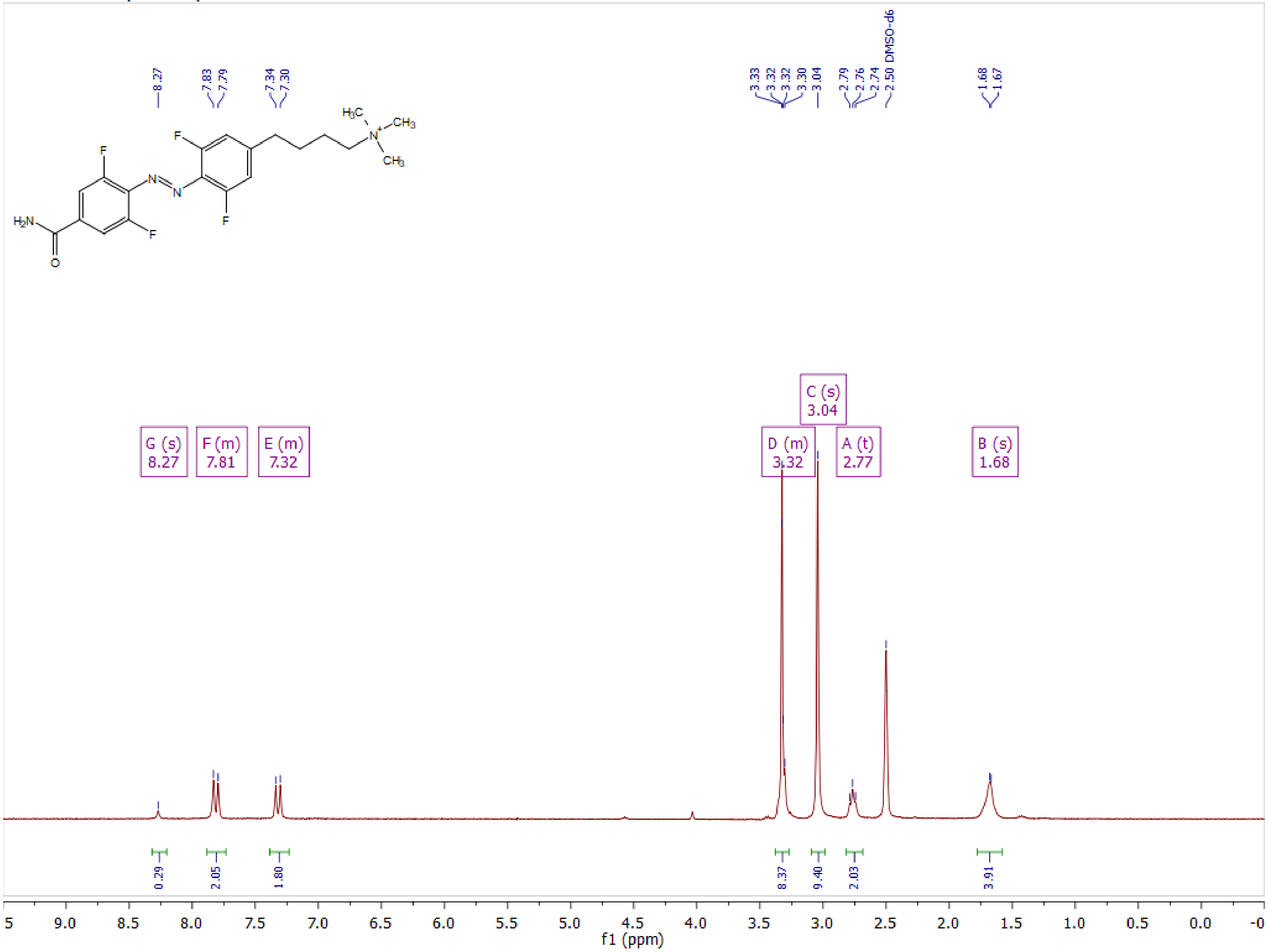

Carbon NMR of 2 (DMSO-d6)

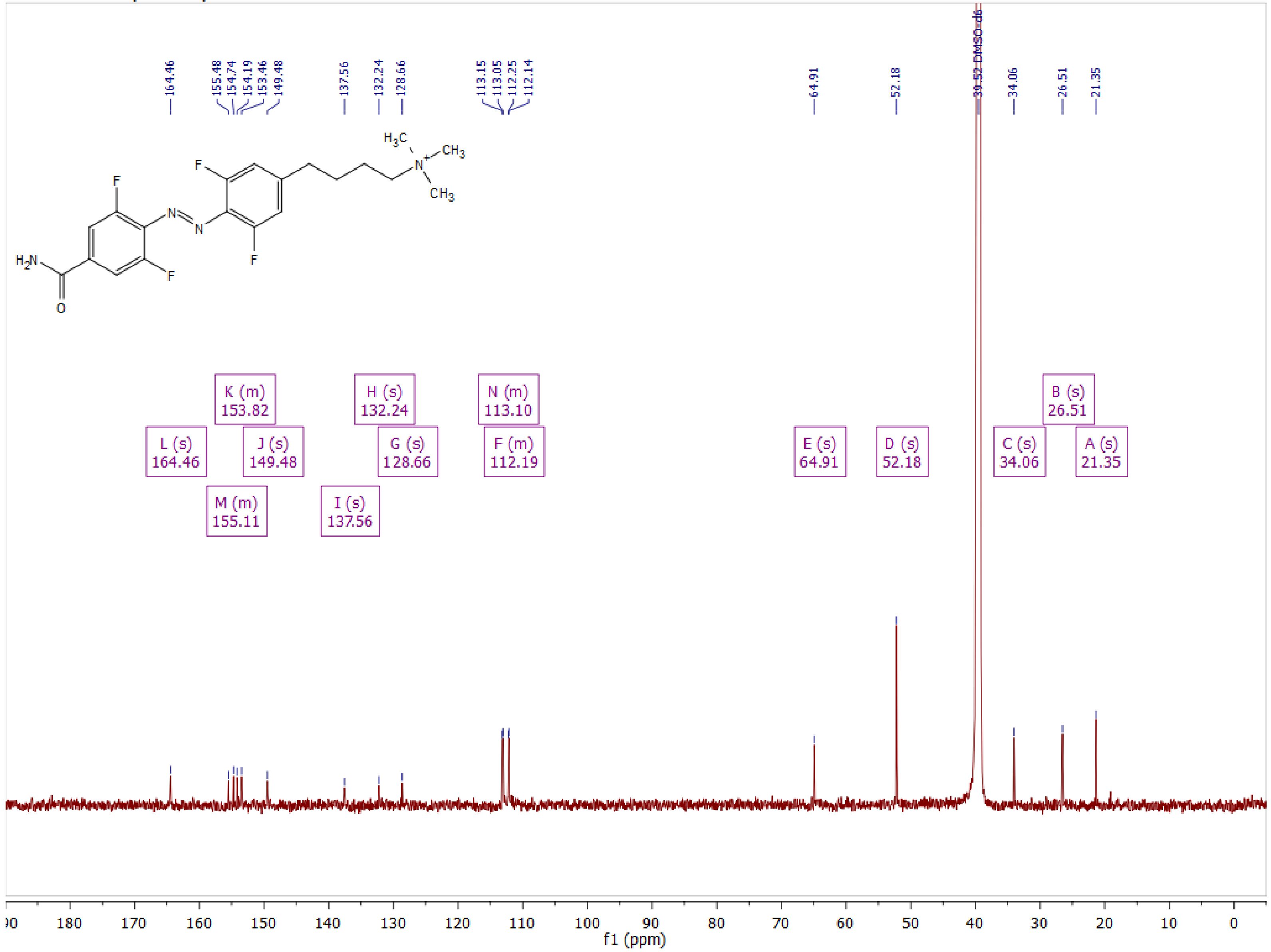

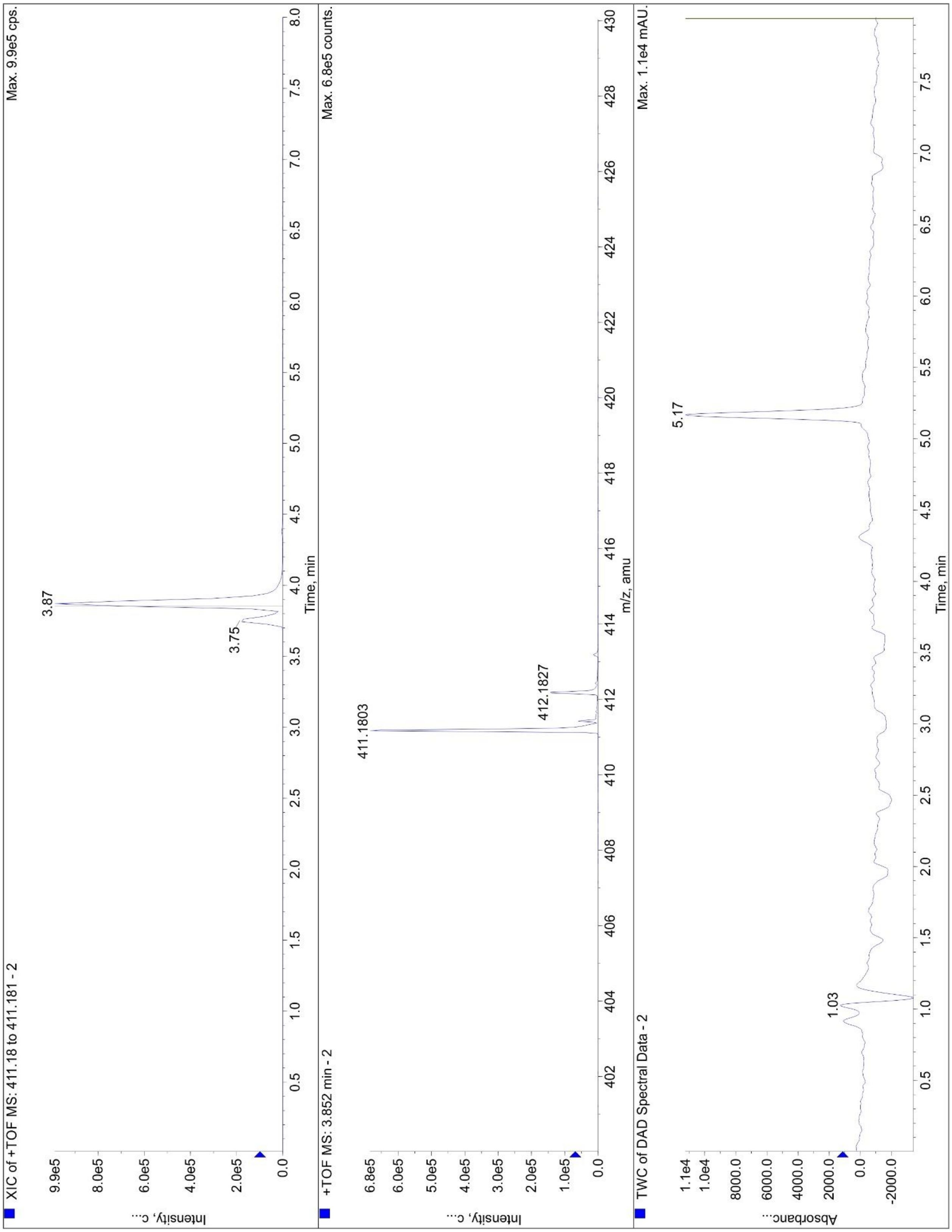
XIC of +TOF MS: 411.18 to 411.181 - 2
Max. 9.9e5 cps.
Intensity, c...
Time, min
3.87
3.75
+TOF MS: 3.852 min - 2
Max. 6.8e5 counts.
m/z, amu
411.1803
412.1827
TWC of DAD Spectral Data - 2
Max. 1.1e4 mAU.
Absorbanc...
5.17
1.03

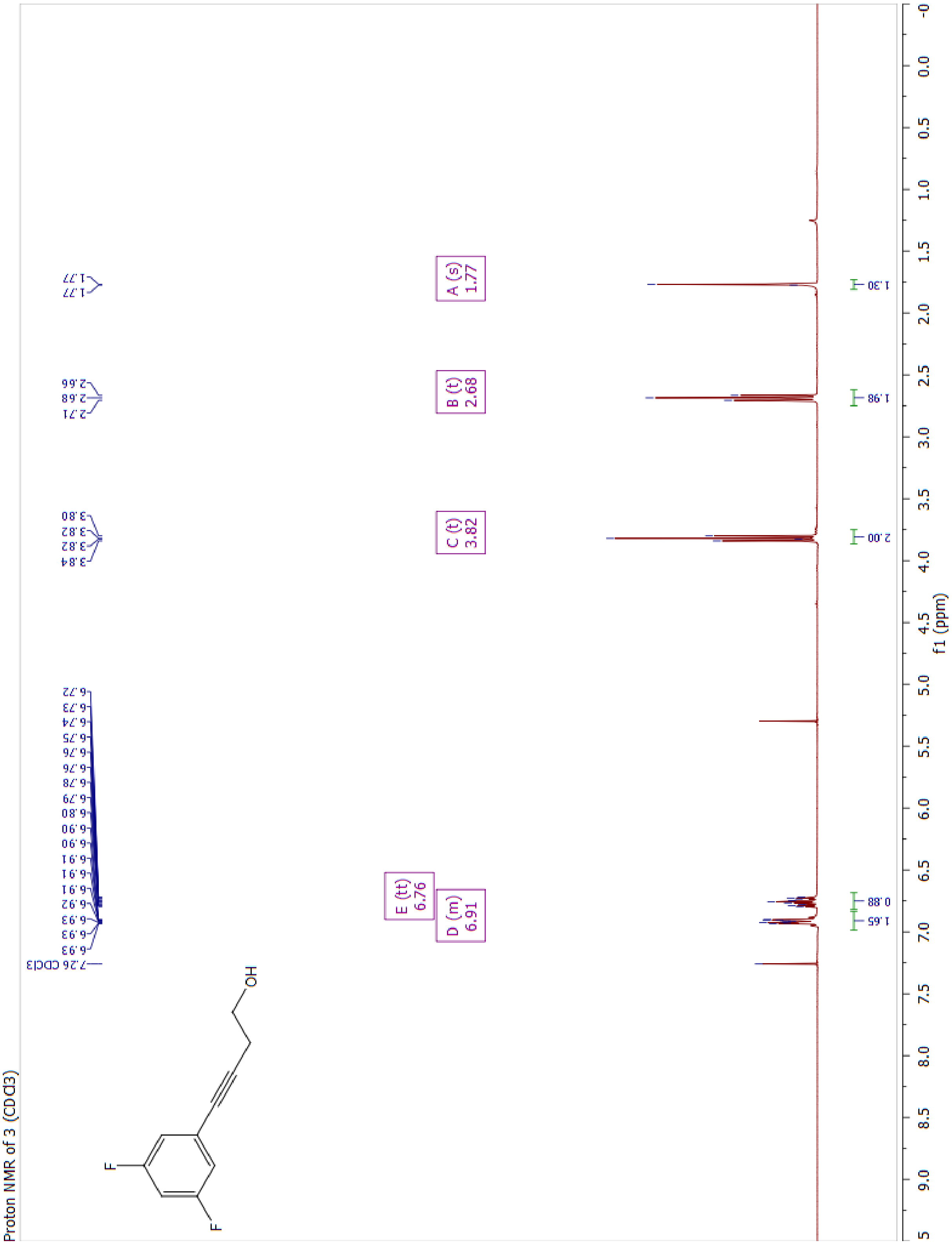
Proton NMR of 3 (CDCl3)
A (s) 1.77
B (t) 2.68
C (t) 3.82
D (m) 6.91
E (tt) 6.76
1.30
1.98
2.00
0.88
1.65
7.26 CDCl3
f1 (ppm)
OH
F
F

Carbon NMR of 3 (CDCl3)

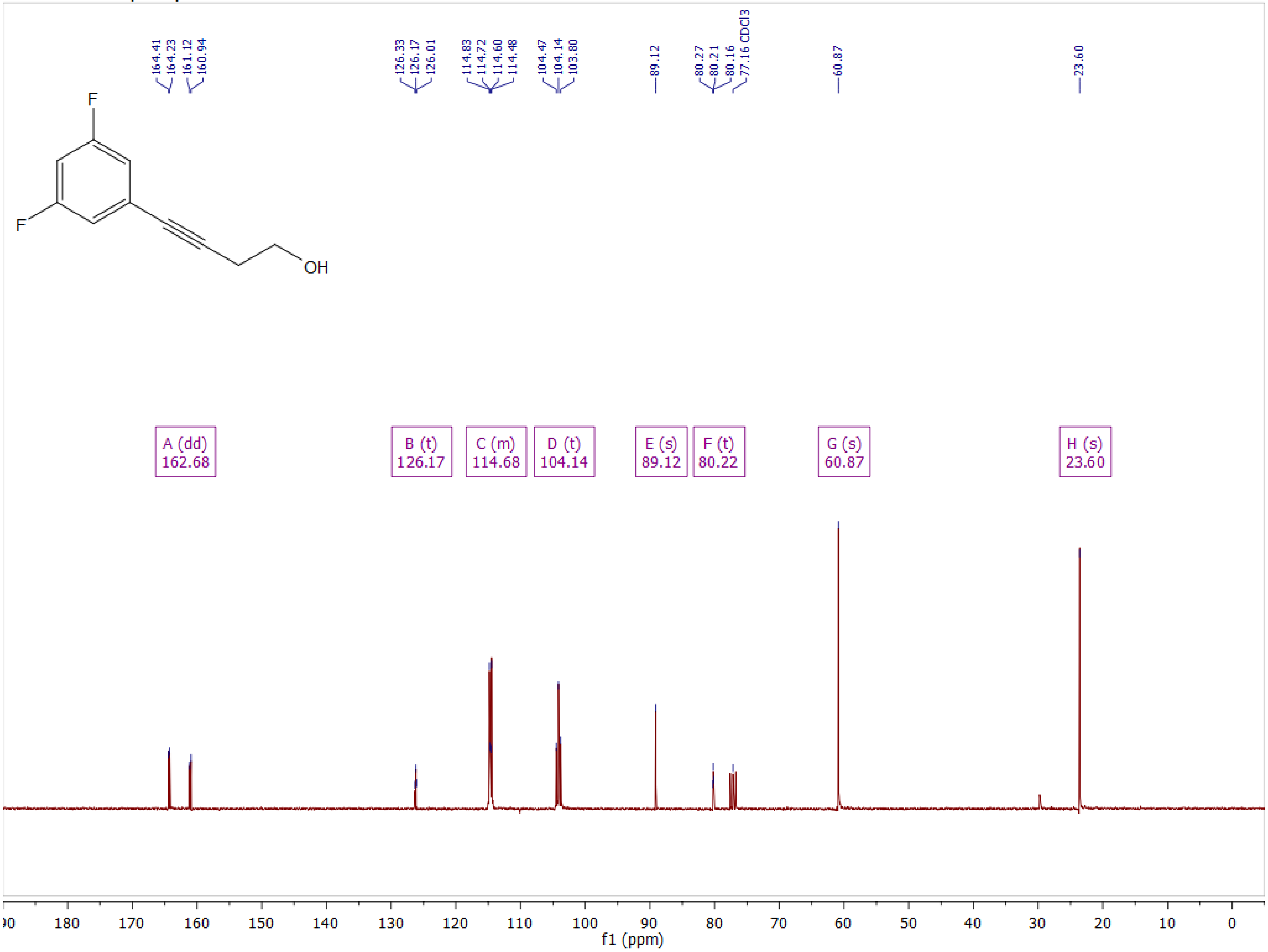

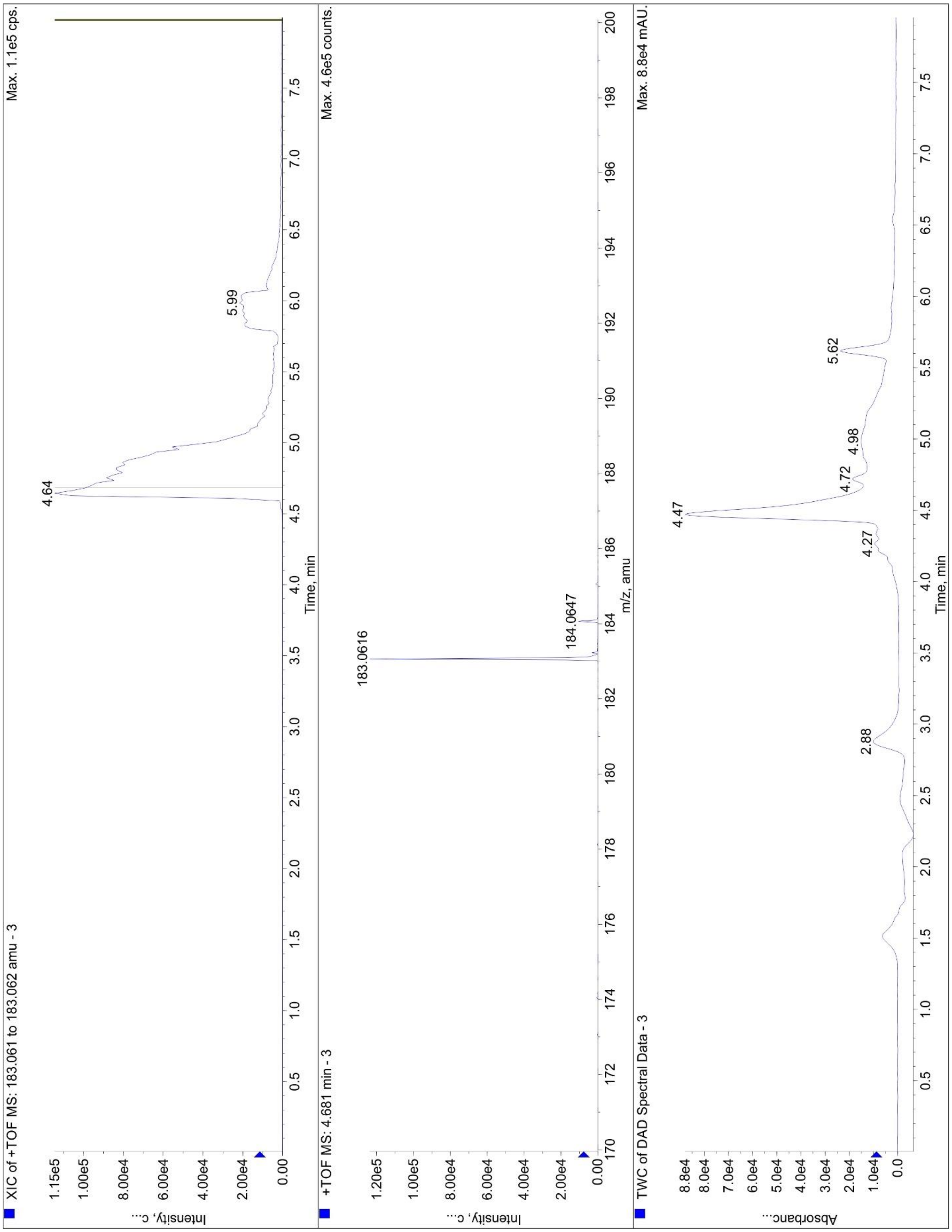
XIC of +TOF MS: 183.061 to 183.062 amu - 3
Max. 1.1e5 cps.
Intensity, c...
Time, min
4.64
5.99
+TOF MS: 4.681 min - 3
Max. 4.6e5 counts.
m/z, amu
183.0616
184.0647
TWC of DAD Spectral Data - 3
Max. 8.8e4 mAU.
Absorbanc...
2.88
4.27
4.47
4.72
4.98
5.62

Proton NMR of 4 (CDCl3)

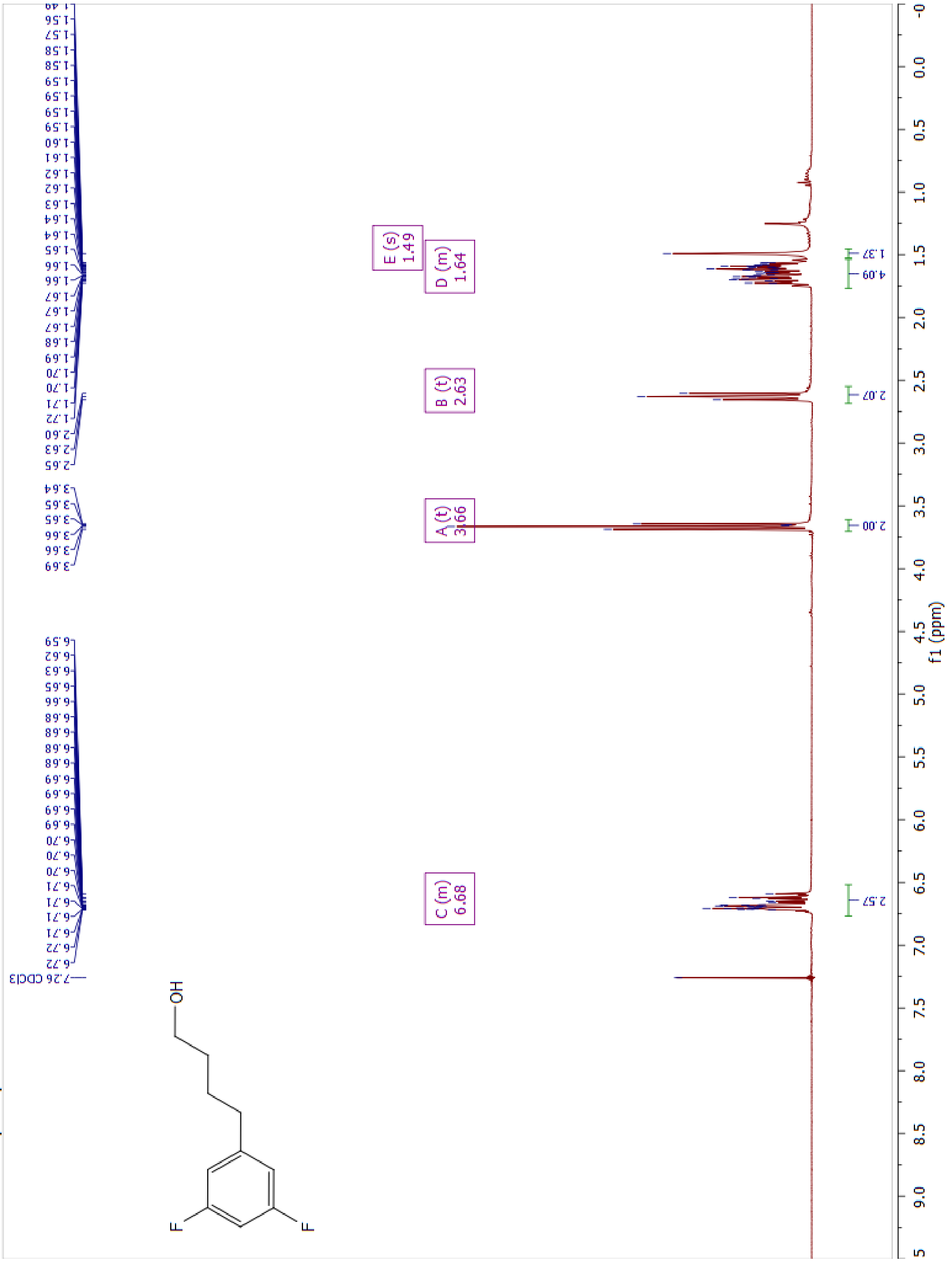

Carbon NMR of 4 (CDCl3)

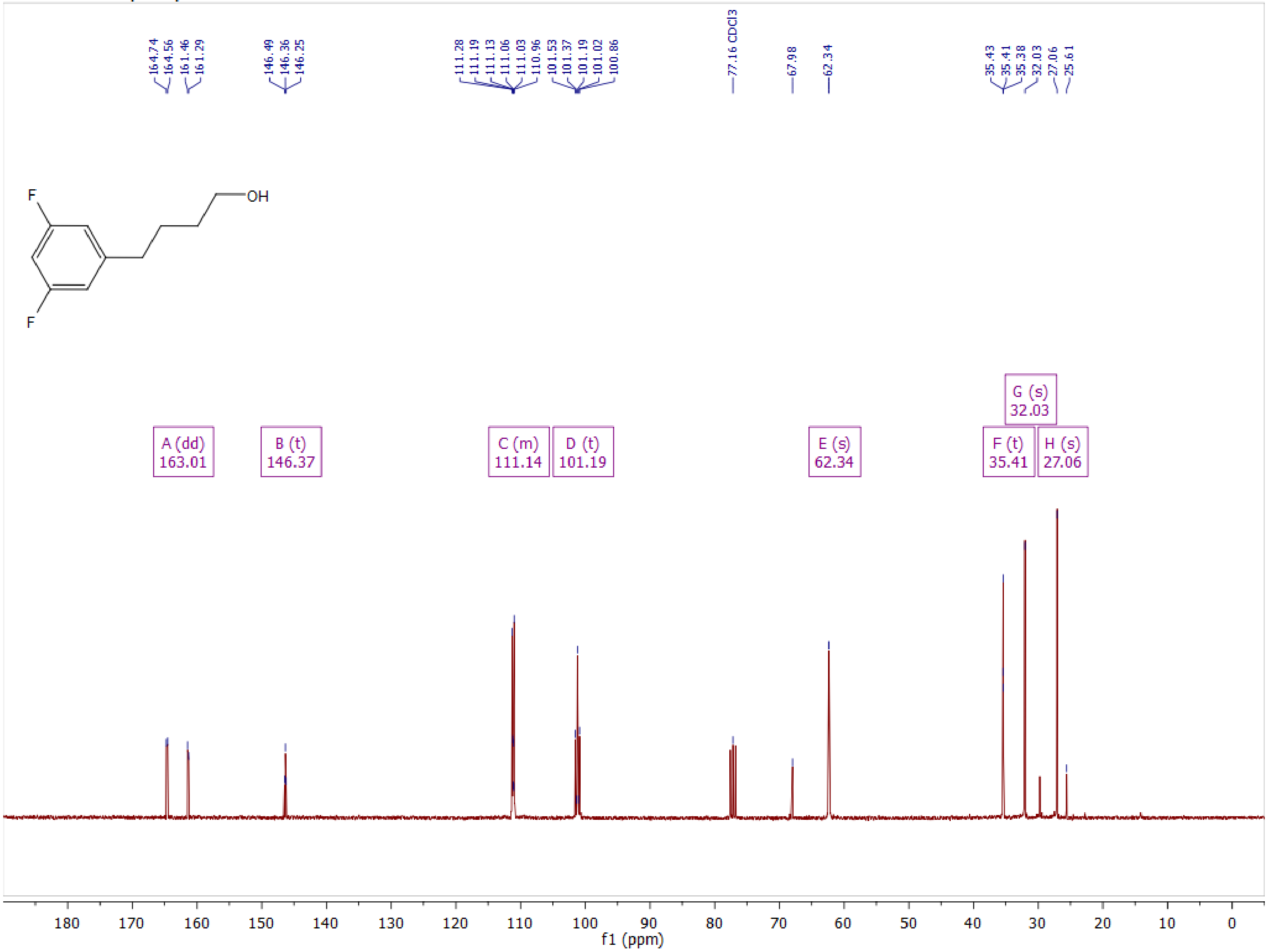

Proton NMR of 5 (CDCl3)

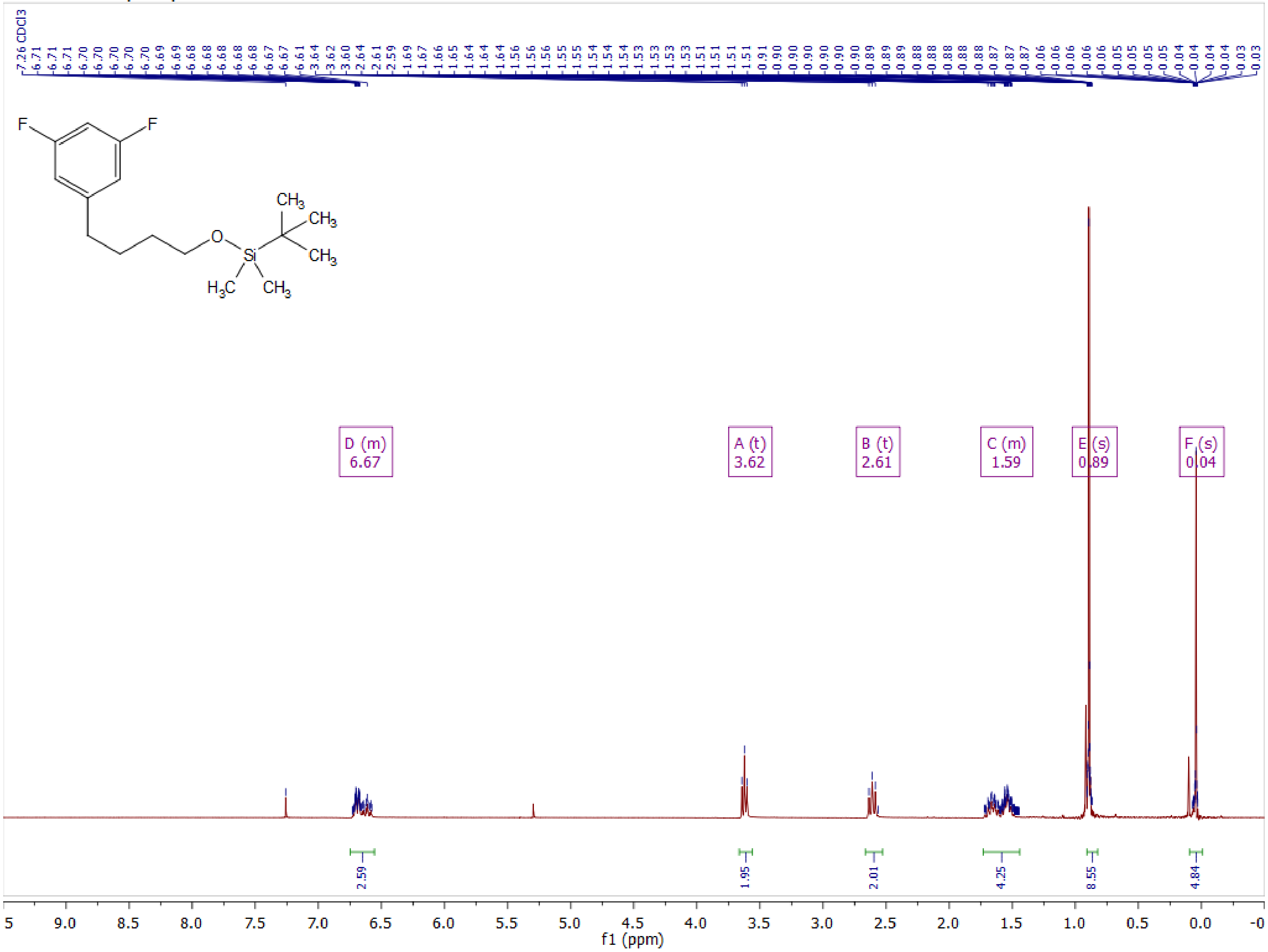

Carbon NMR of 5 (CDCl3)

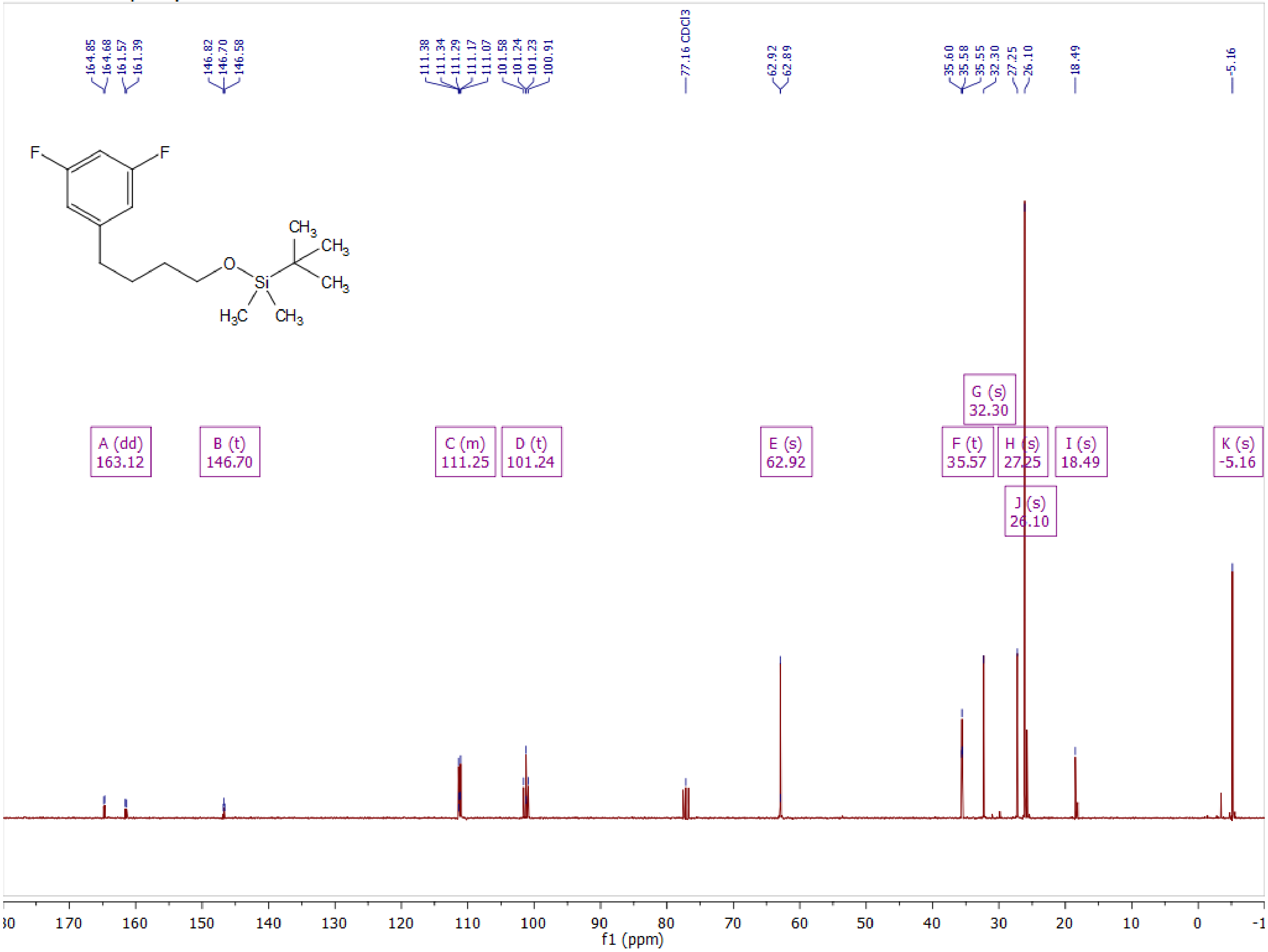

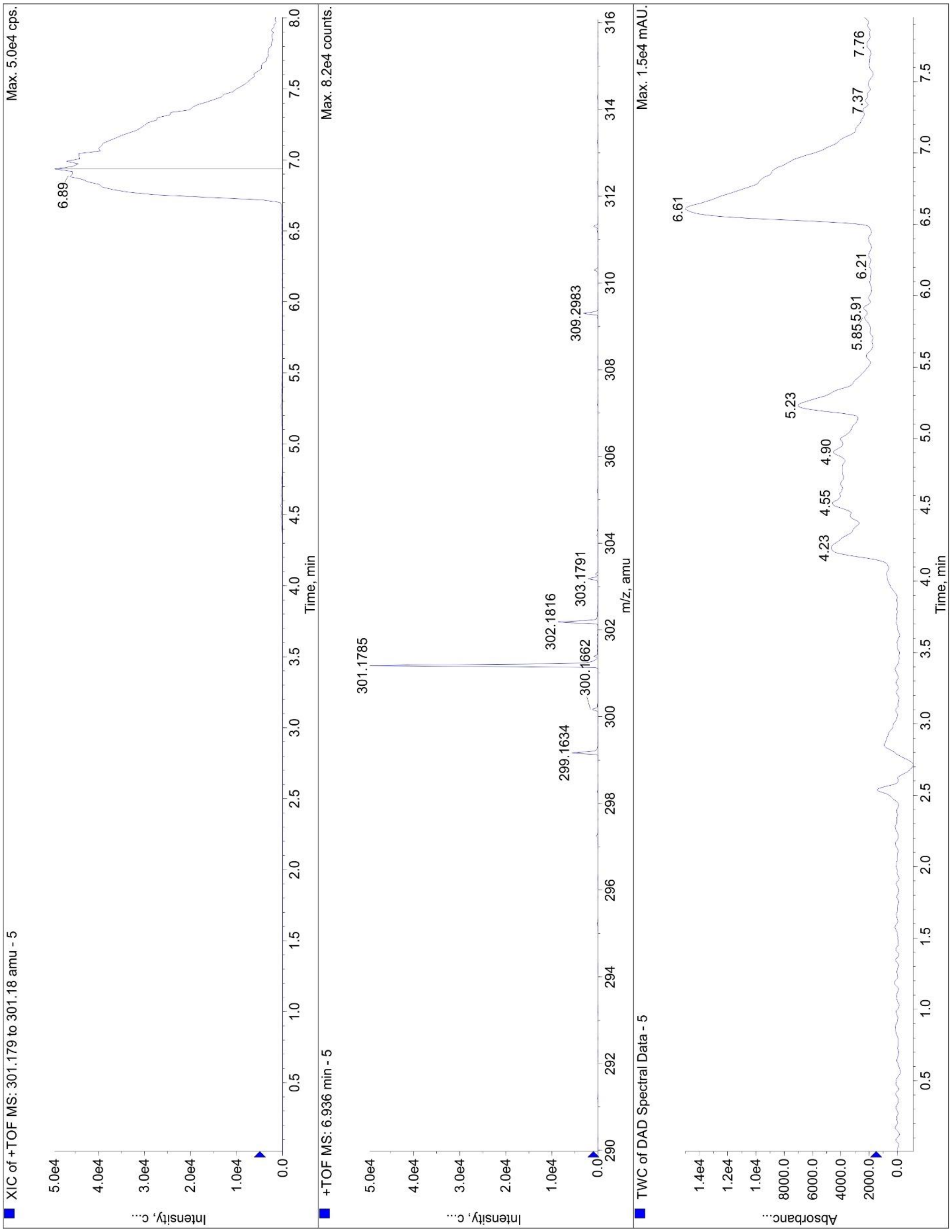

XIC of +TOF MS: 301.179 to 301.18 amu - 5
Max. 5.0e4 cps.
Intensity, c...
Time, min
6.89
+TOF MS: 6.936 min - 5
Max. 8.2e4 counts.
m/z, amu
301.1785
302.1816
303.1791
300.1662
299.1634
309.2983
TWC of DAD Spectral Data - 5
Max. 1.5e4 mAU.
Absorbanc...
6.61
5.23
4.23
4.55
4.90
5.85
5.91
6.21
7.37
7.76

Proton NMR of 6 (CDCl3)

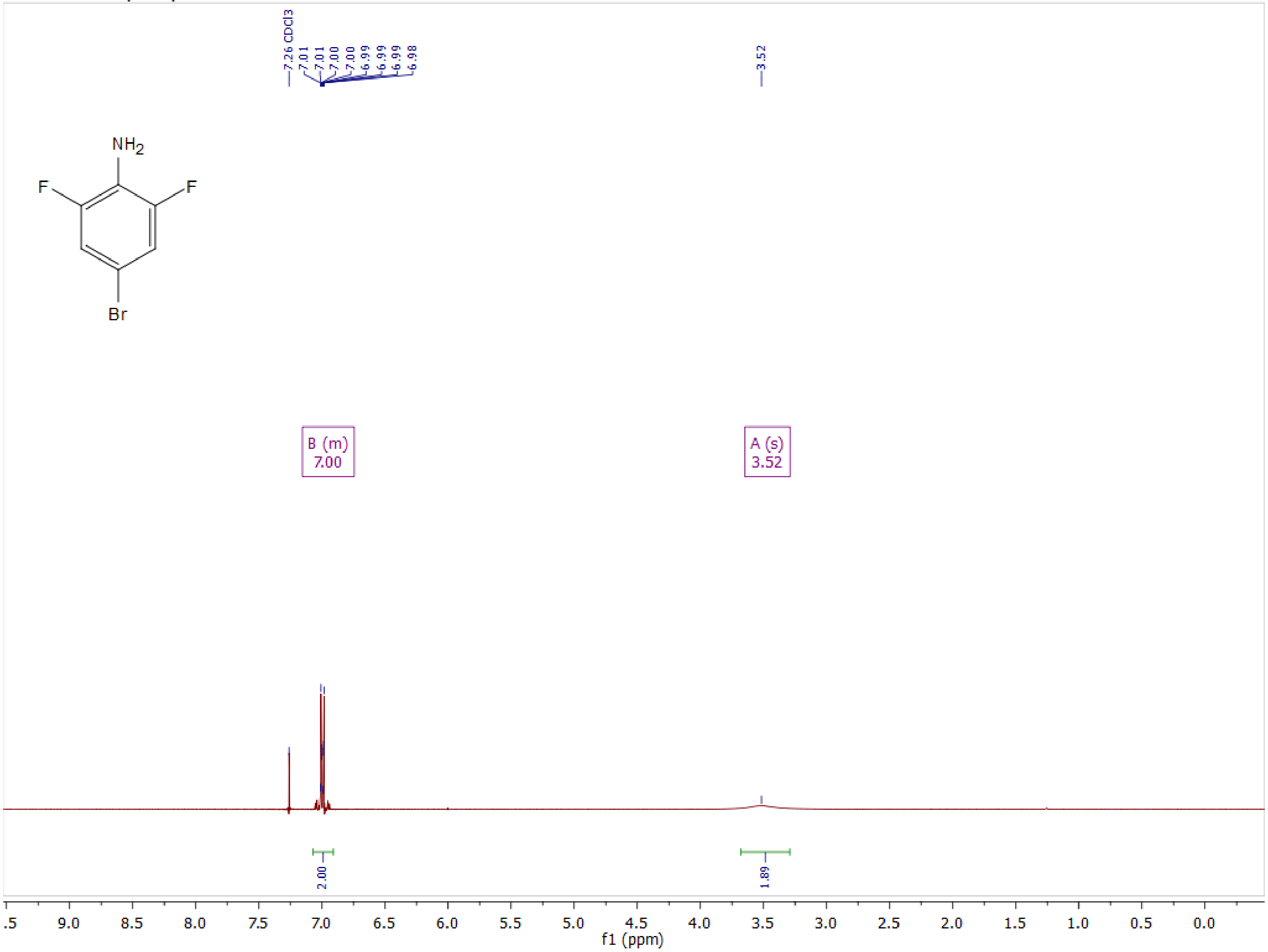

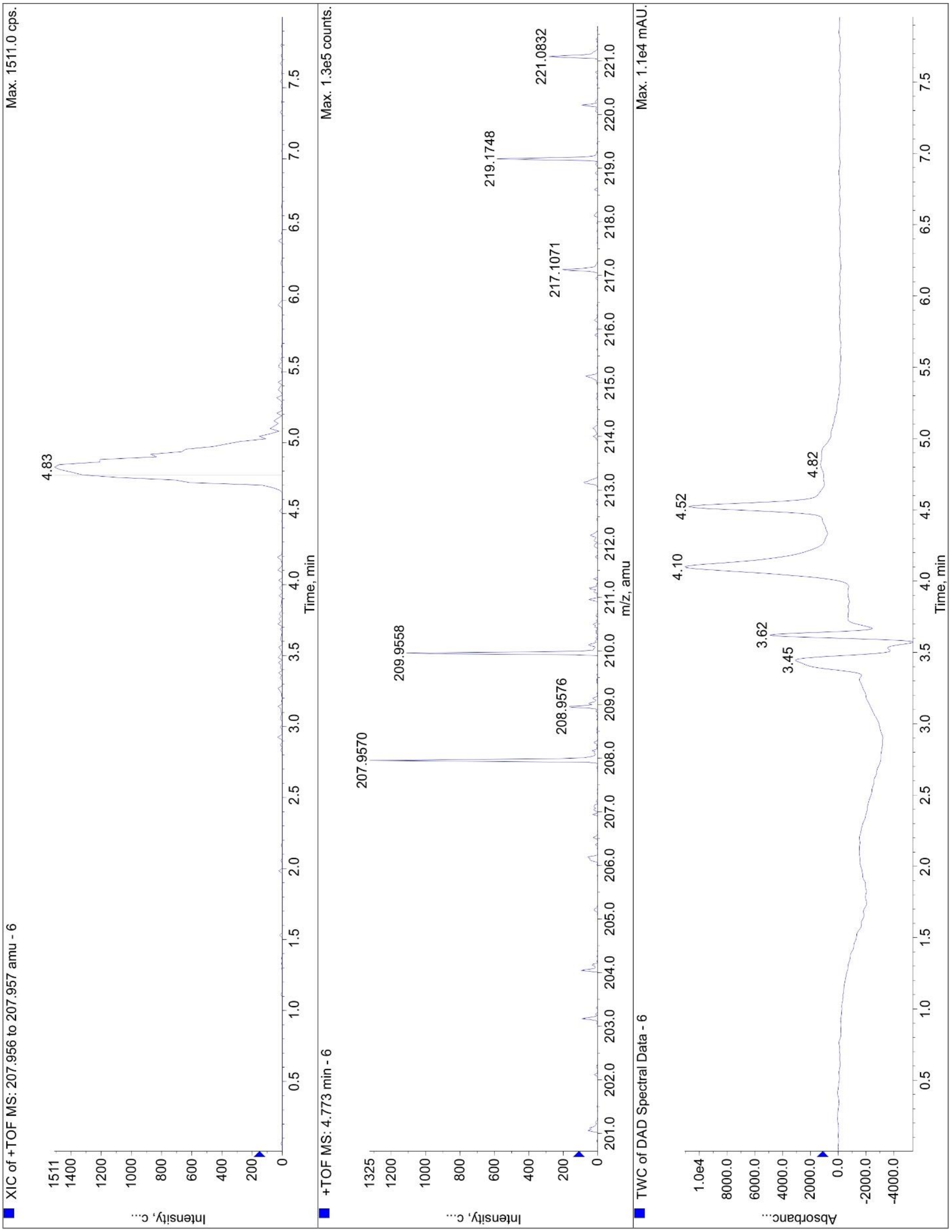

XIC of +TOF MS: 207.956 to 207.957 amu - 6
Max. 1511.0 cps.
Intensity, c...
4.83
Time, min
+TOF MS: 4.773 min - 6
Max. 1.3e5 counts.
207.9570
208.9576
209.9558
217.1071
219.1748
221.0832
m/z, amu
TWC of DAD Spectral Data - 6
Max. 1.1e4 mAU.
Absorbanc...
3.45
3.62
4.10
4.52
4.82
Time, min

Proton NMR of 7 (CDCl3)

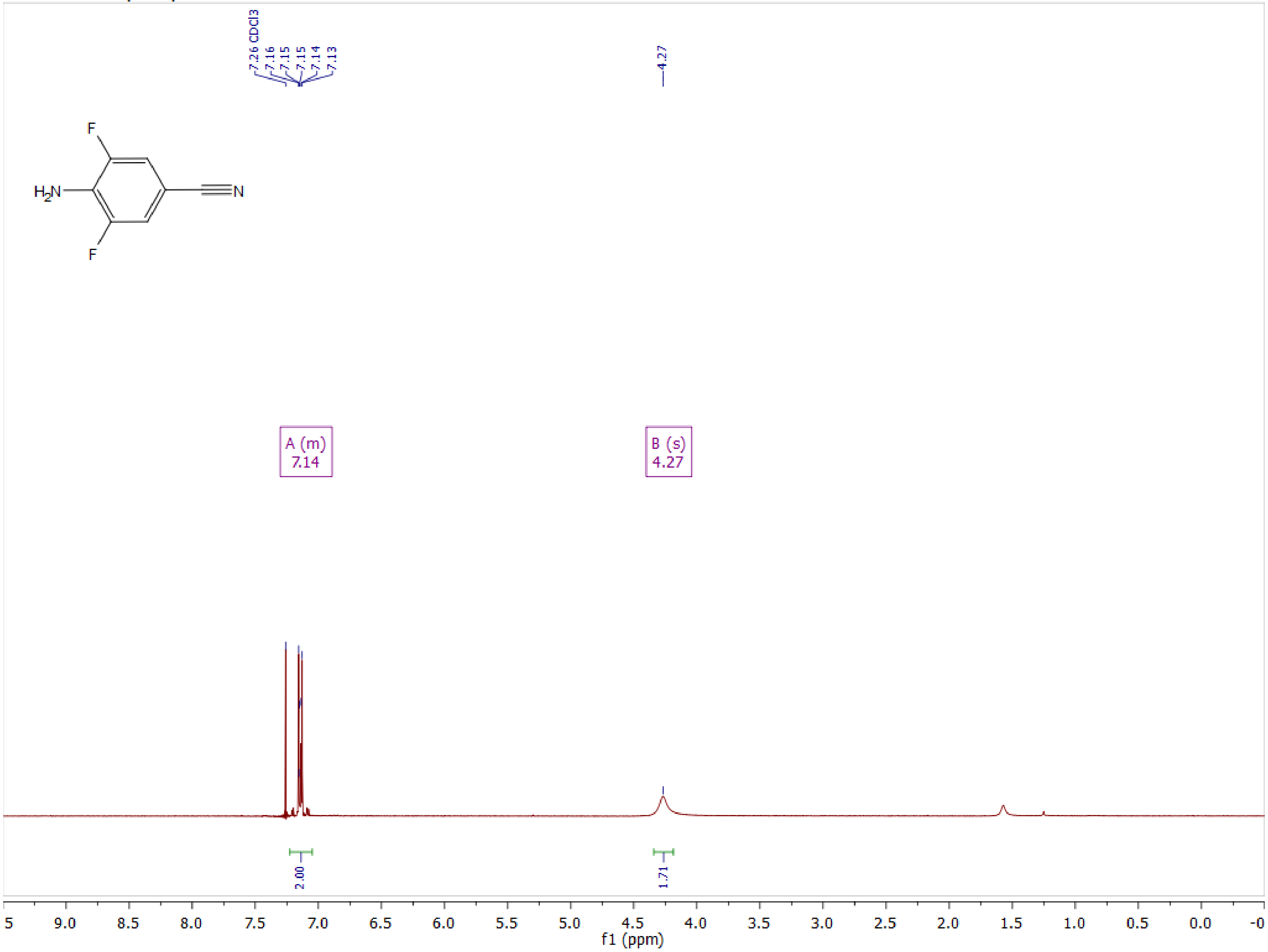

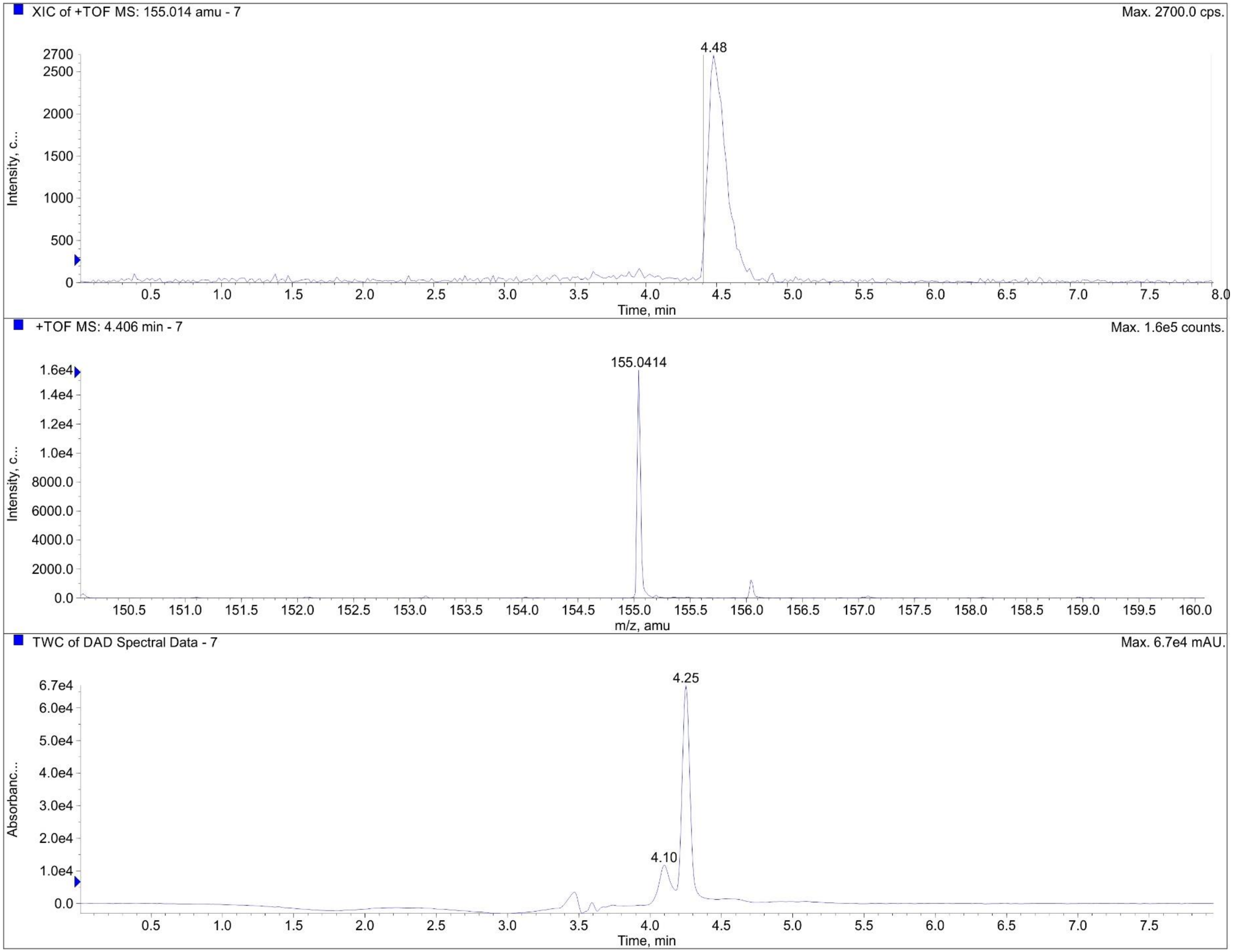
XIC of +TOF MS: 155.014 amu - 7
Max. 2700.0 cps.
4.48
Intensity, c...
Time, min
+TOF MS: 4.406 min - 7
Max. 1.6e5 counts.
155.0414
Intensity, c...
m/z, amu
TWC of DAD Spectral Data - 7
Max. 6.7e4 mAU.
4.25
4.10
Absorbanc...
Time, min

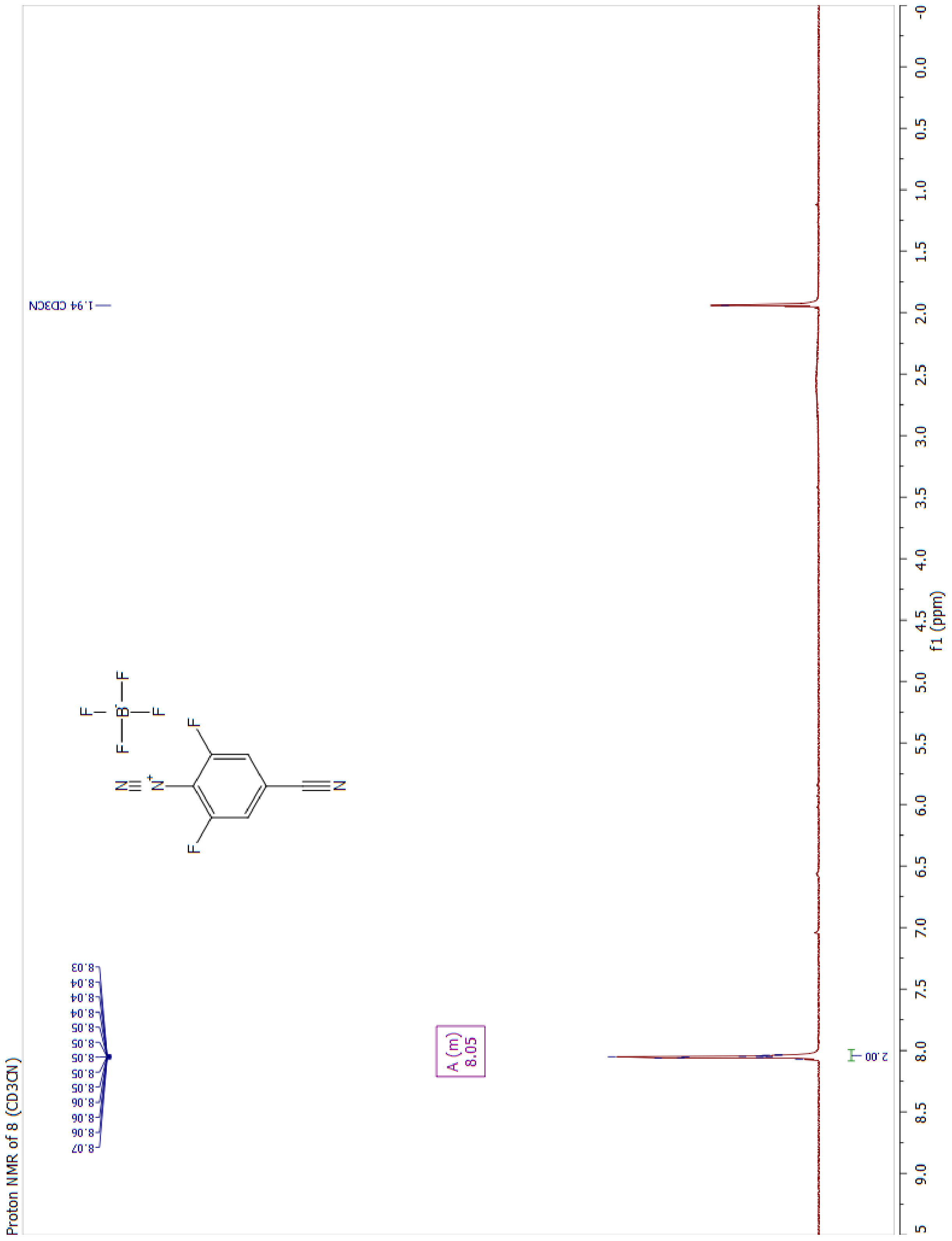

Proton NMR of 8 (CD3CN)
8.03
8.04
8.04
8.04
8.05
8.05
8.05
8.05
8.05
8.06
8.06
8.06
8.07
1.94 CD3CN
A (m)
8.05
2.00
f1 (ppm)

Proton NMR of 9 (CDCl3)

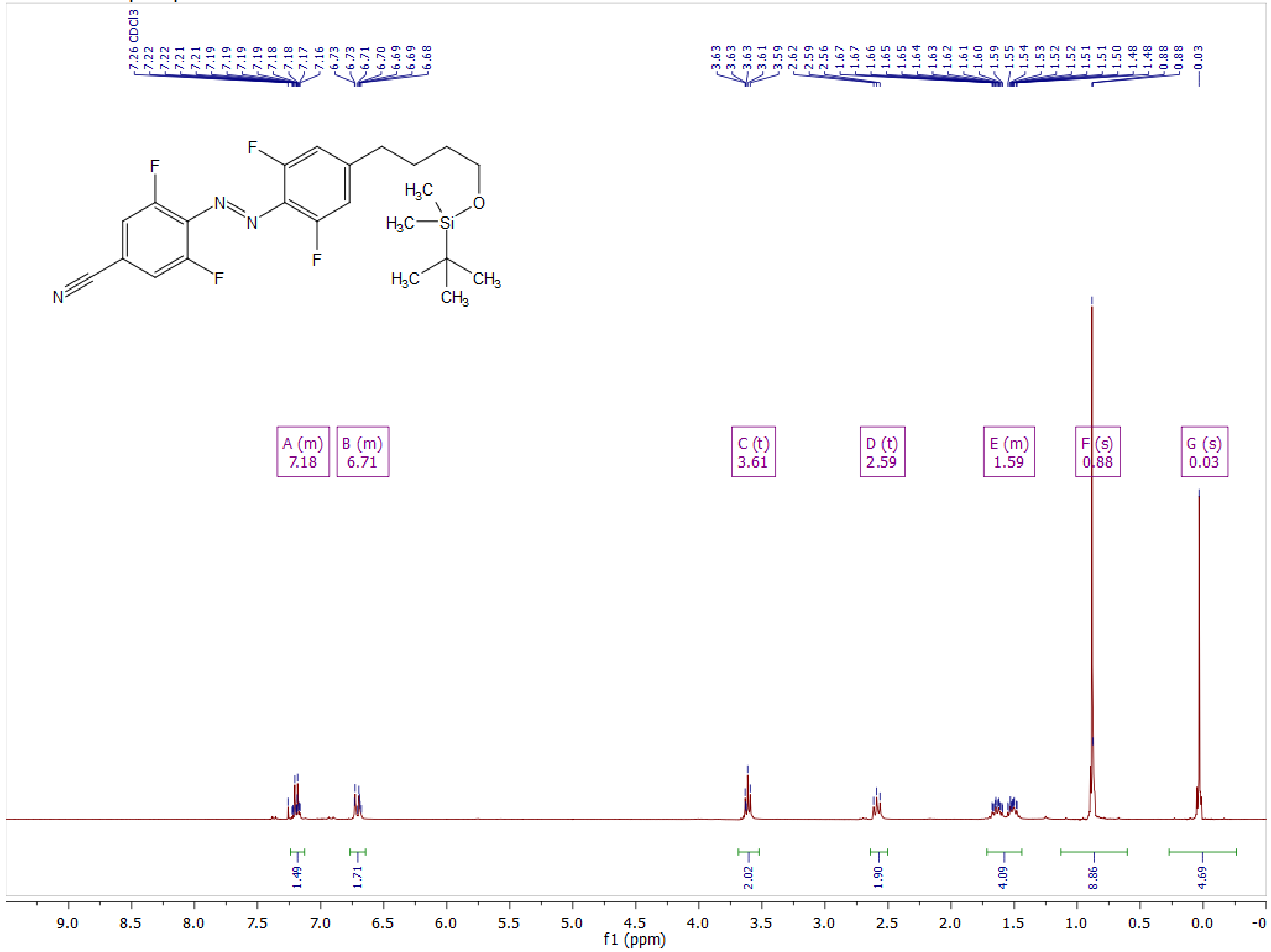

Carbon NMR of 9 (CDCl3)

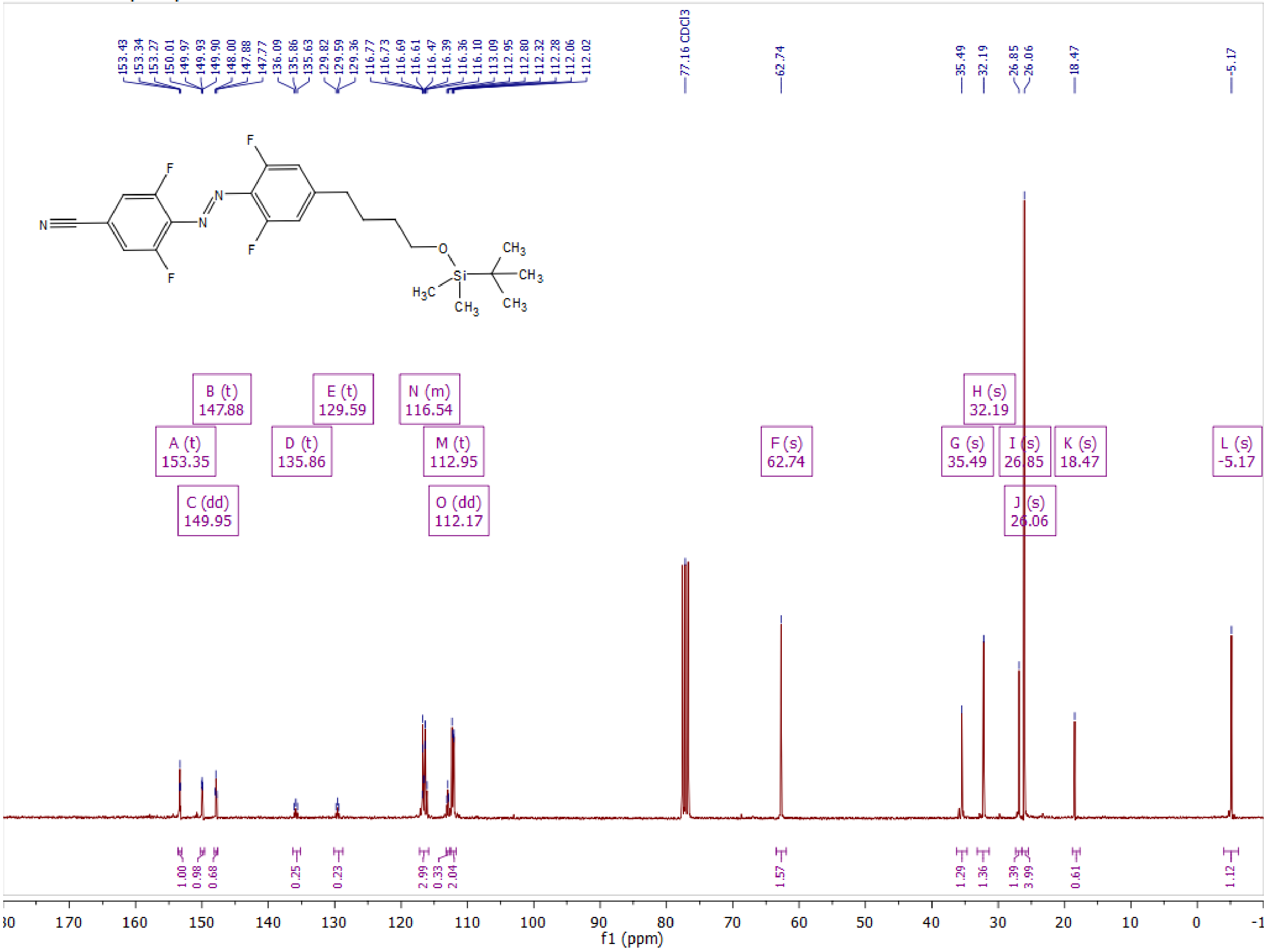

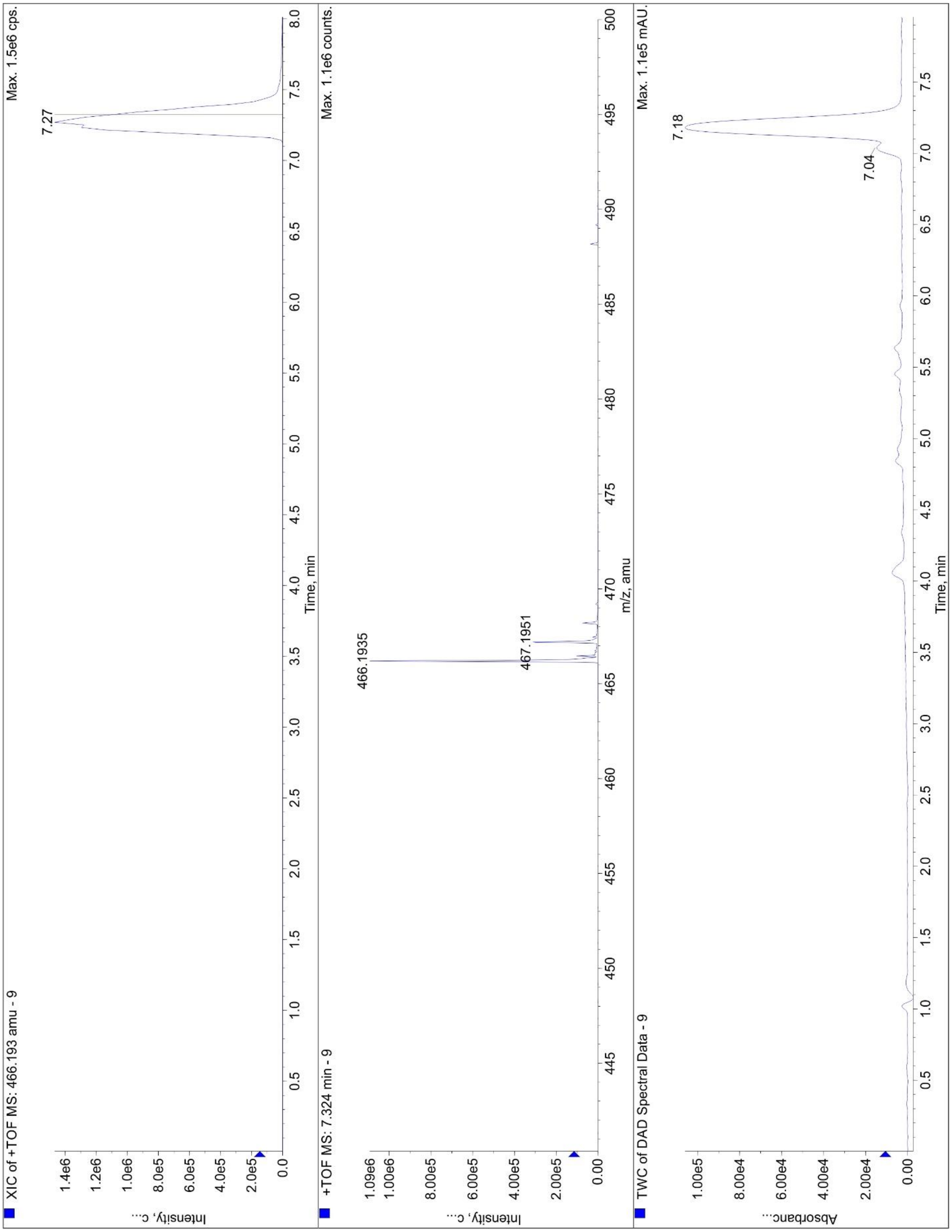
XIC of +TOF MS: 466.193 amu - 9
Max. 1.5e6 cps.
Intensity, c...
Time, min
7.27
+TOF MS: 7.324 min - 9
Max. 1.1e6 counts.
Intensity, c...
m/z, amu
466.1935
467.1951
TWC of DAD Spectral Data - 9
Max. 1.1e5 mAU.
Absorbanc...
Time, min
7.18
7.04

Proton NMR of 10 (CDCl3)

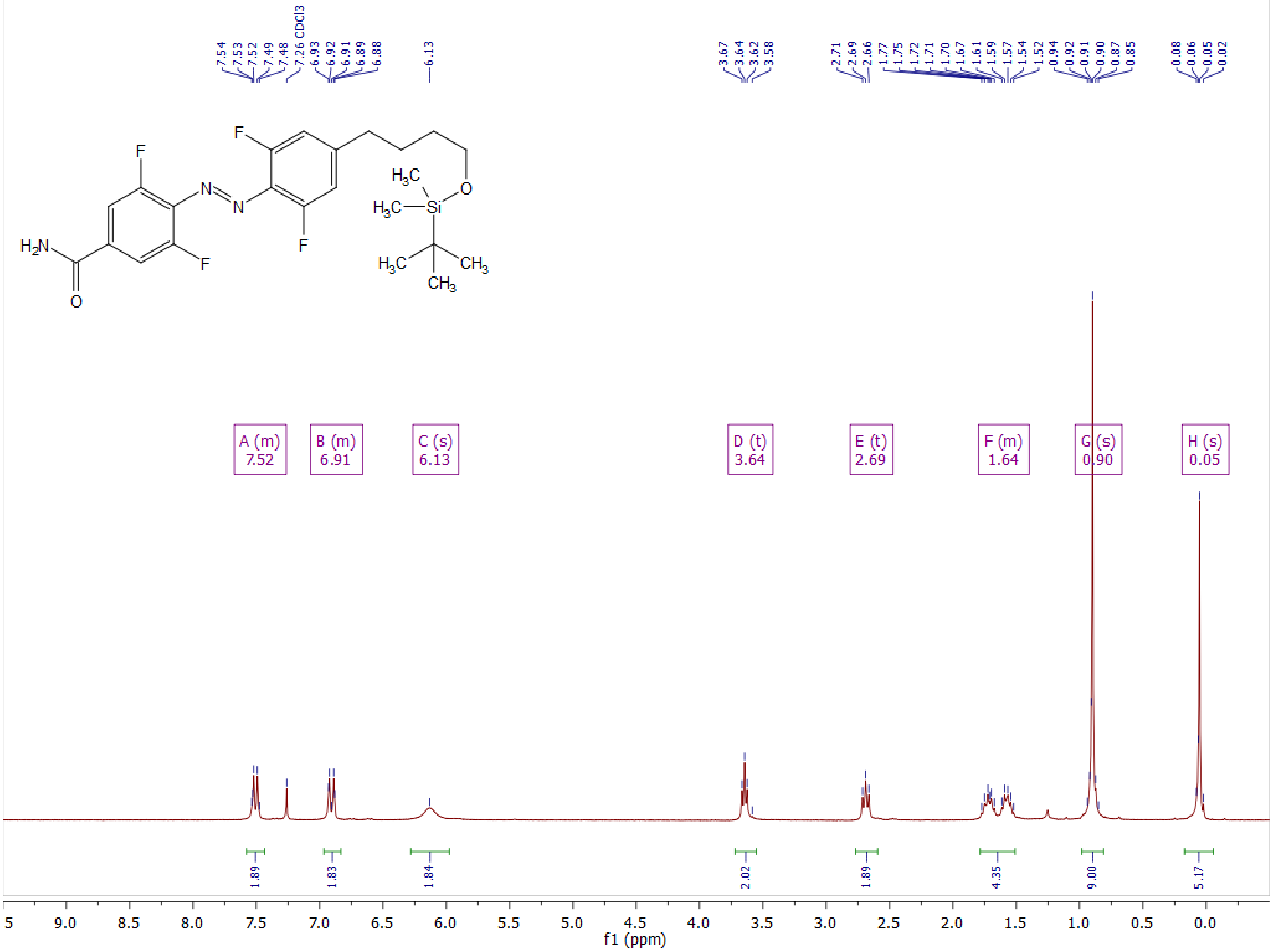

Carbon NMR of 10 (CDCl3)

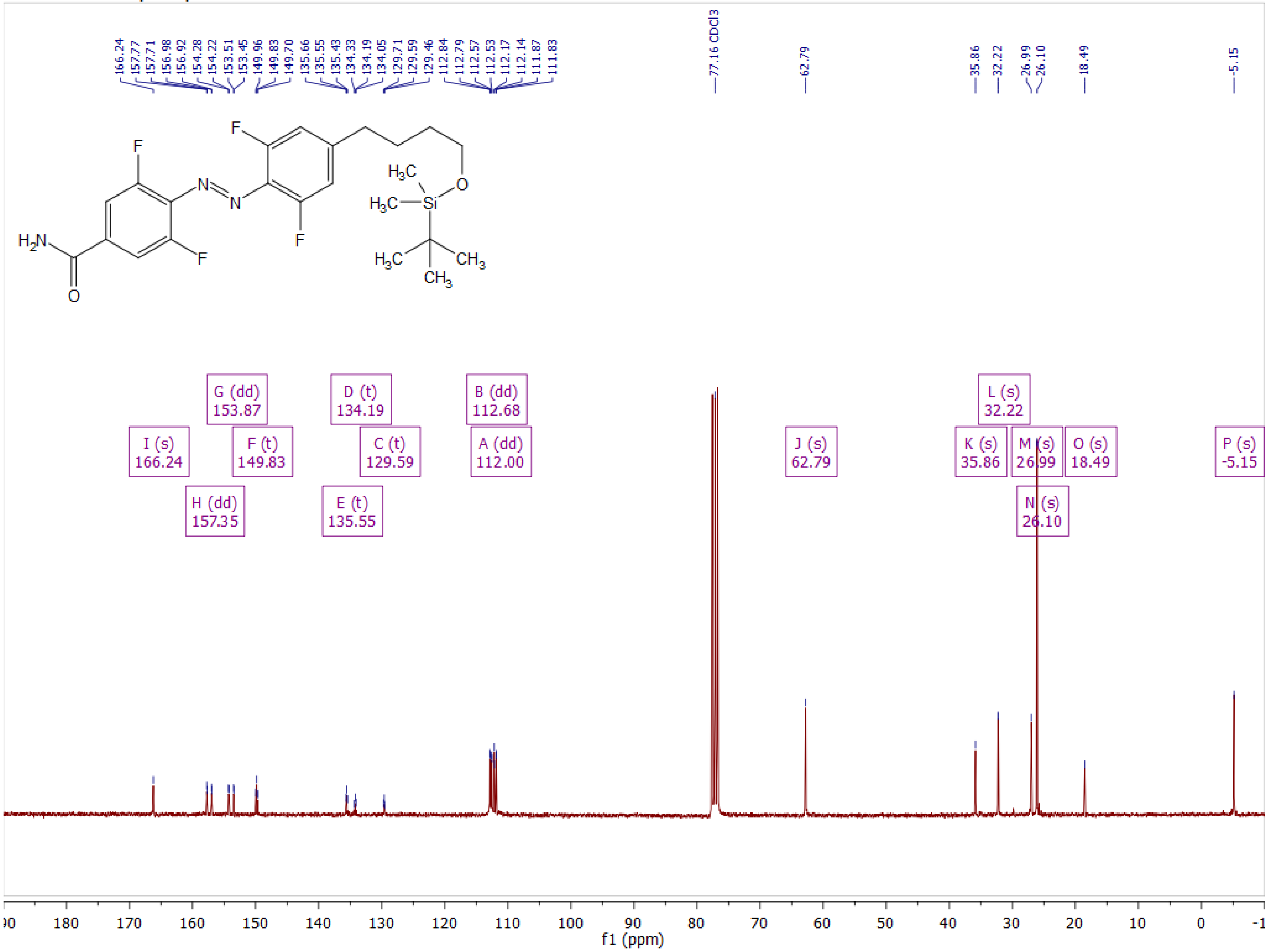

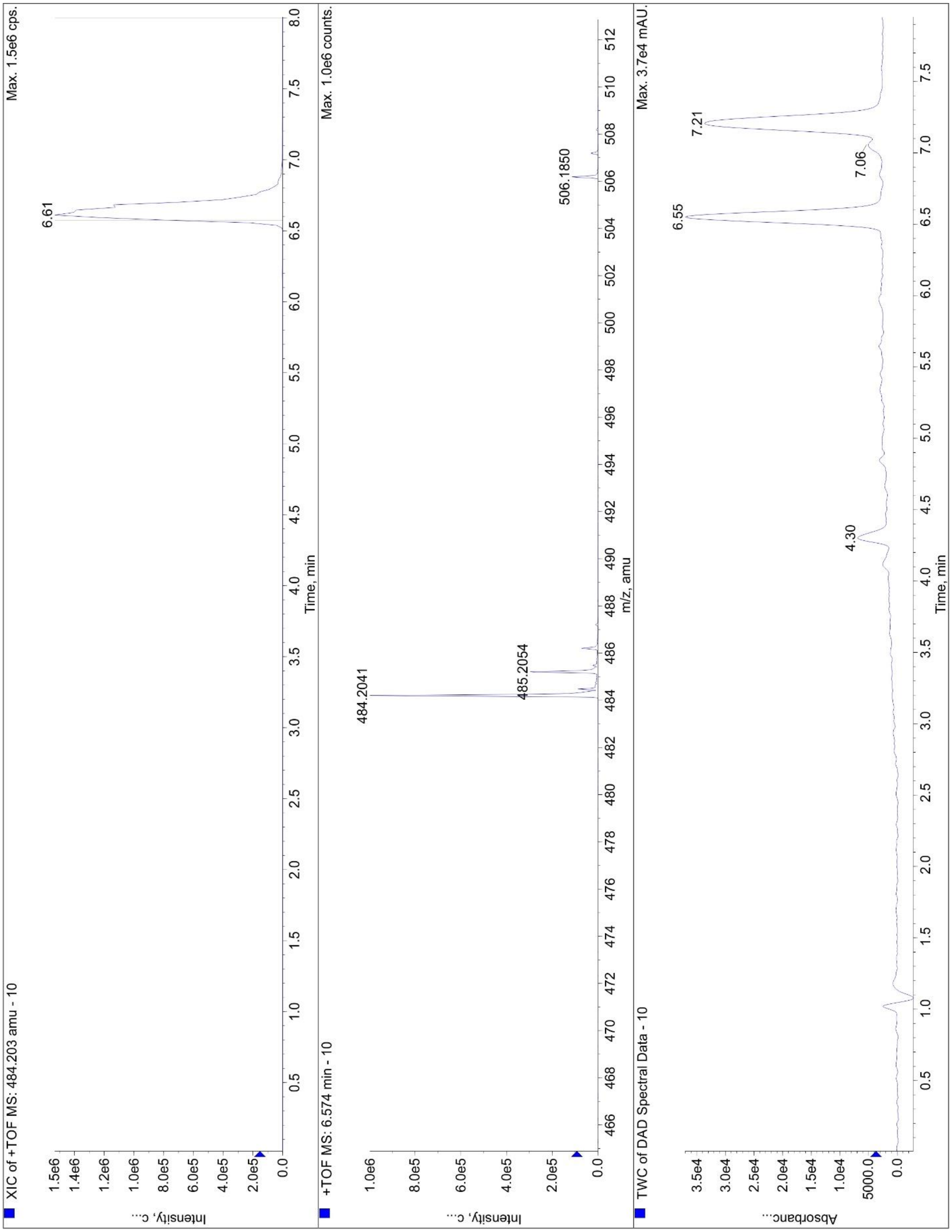
XIC of +TOF MS: 484.203 amu - 10
Max. 1.5e6 cps.
Intensity, c...
Time, min
6.61
+TOF MS: 6.574 min - 10
Max. 1.0e6 counts.
m/z, amu
484.2041
485.2054
506.1850
TWC of DAD Spectral Data - 10
Max. 3.7e4 mAU.
Absorbanc...
4.30
6.55
7.06
7.21

Proton NMR of 11 (DMSO-d6)

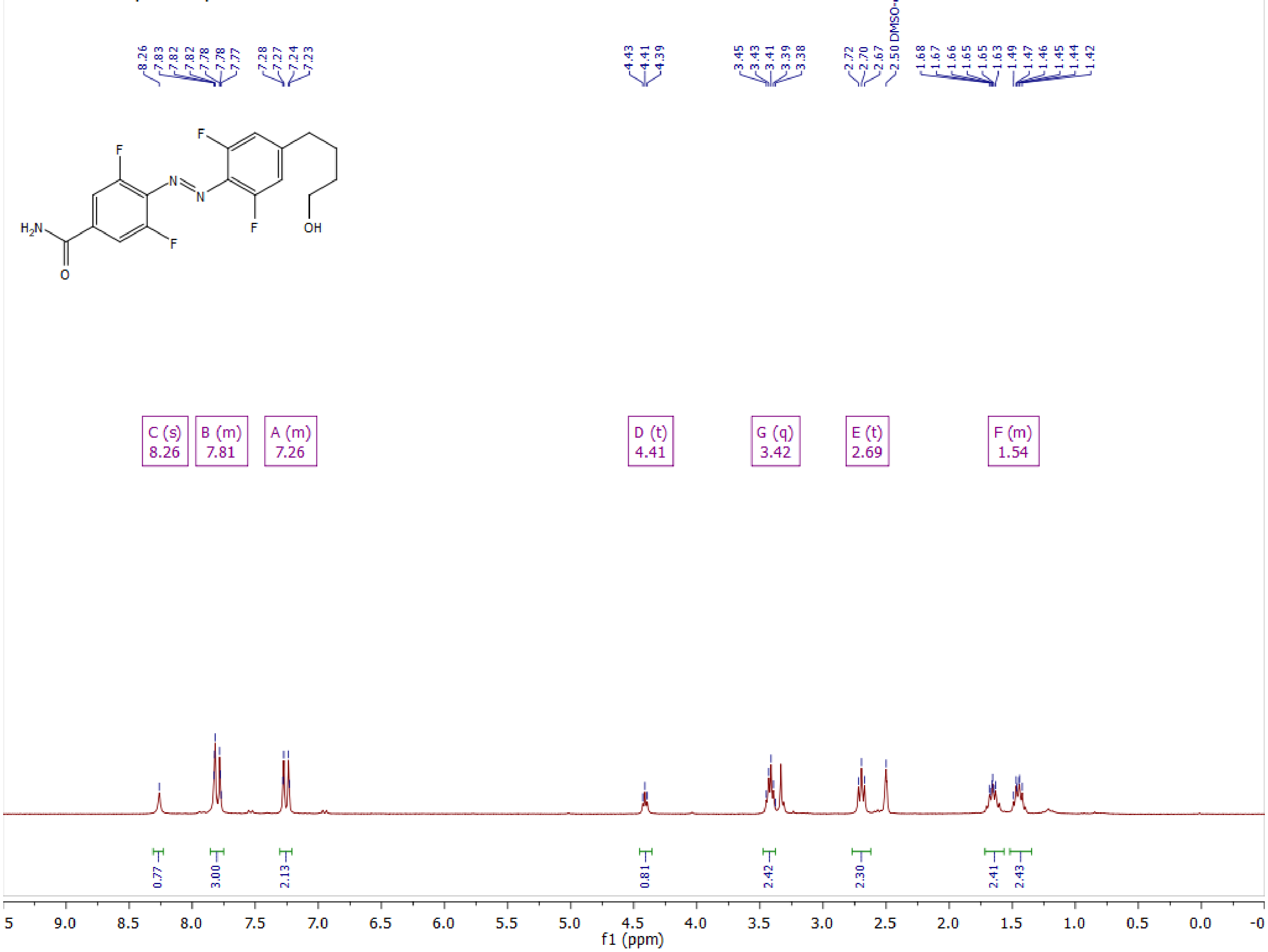

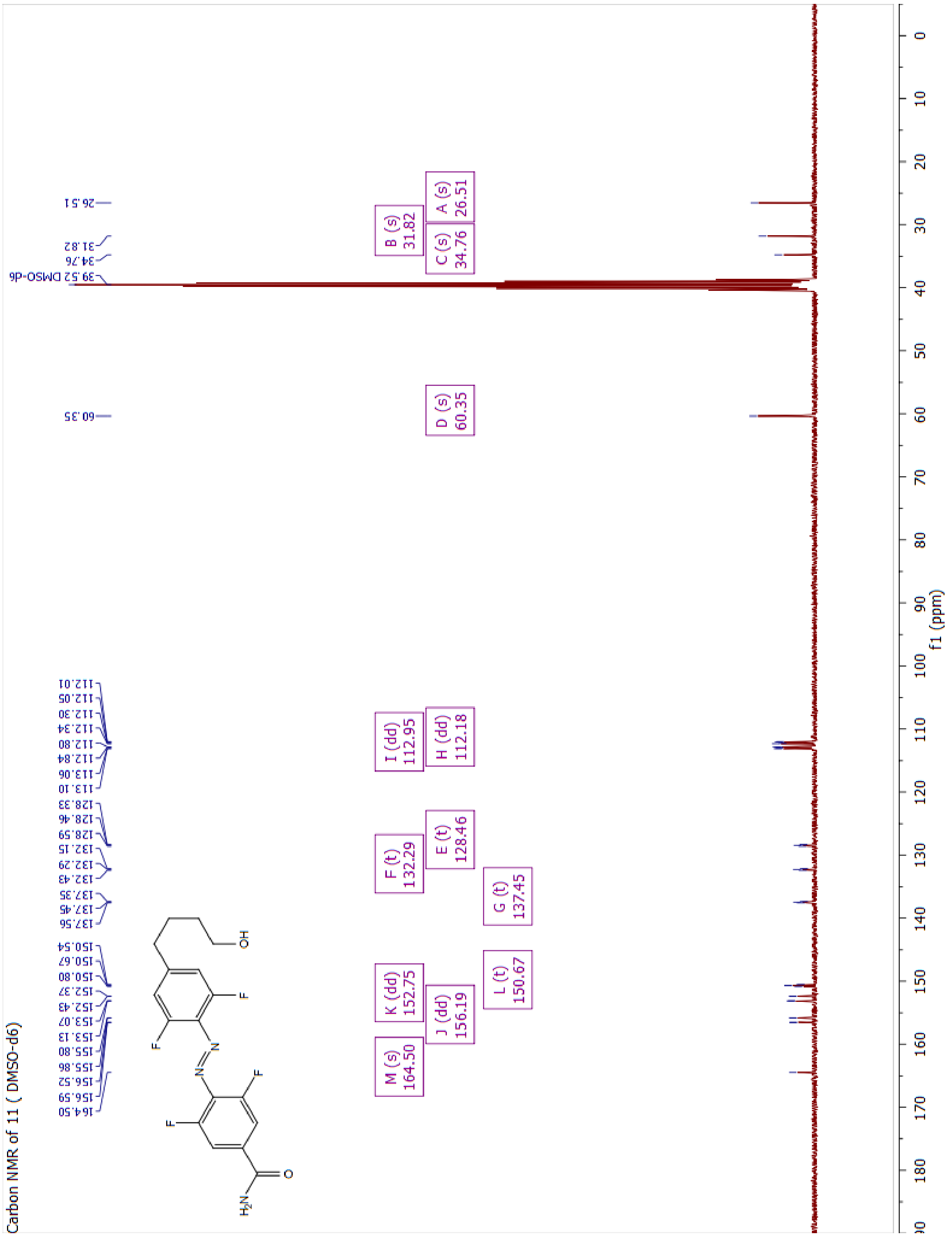

Carbon NMR of 11 (DMSO-d6)
26.51
31.82
34.76
39.52 DMSO-d6
60.35
112.01
112.05
112.30
112.34
112.80
112.84
113.06
113.10
128.33
128.46
128.59
132.15
132.29
132.43
137.35
137.45
137.56
150.54
150.67
150.80
152.37
152.43
153.07
153.13
155.80
155.86
156.52
156.59
164.50
A (s) 26.51
B (s) 31.82
C (s) 34.76
D (s) 60.35
E (t) 128.46
F (t) 132.29
G (t) 137.45
H (dd) 112.18
I (dd) 112.95
J (dd) 156.19
K (dd) 152.75
L (t) 150.67
M (s) 164.50
f1 (ppm)

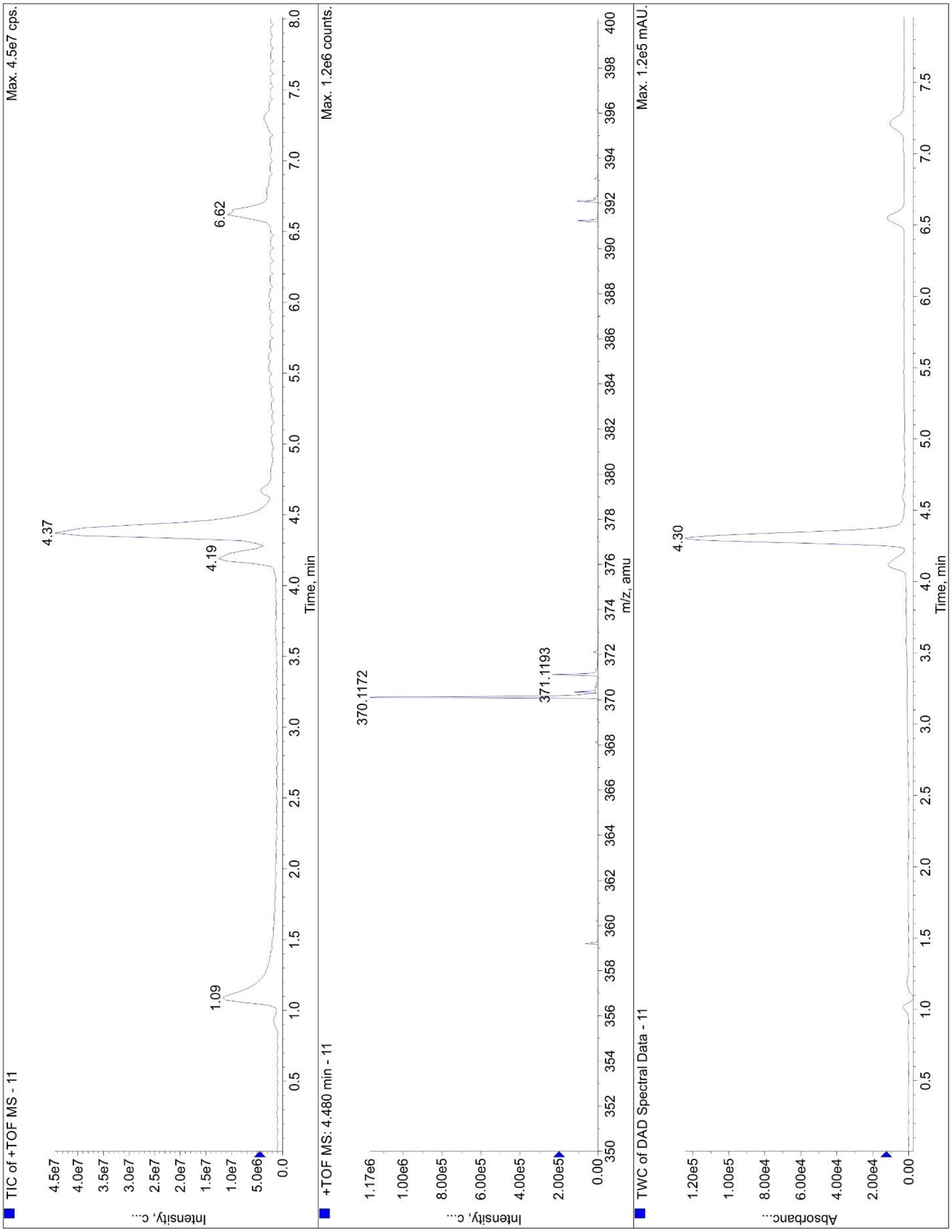
TIC of +TOF MS - 11
Max. 4.5e7 cps.
Intensity, c...
Time, min
1.09
4.19
4.37
6.62
+TOF MS: 4.480 min - 11
Max. 1.2e6 counts.
m/z, amu
370.1172
371.1193
TWC of DAD Spectral Data - 11
Max. 1.2e5 mAU.
Absorbanc...
4.30

Proton NMR of 14 (DMSO-d6)

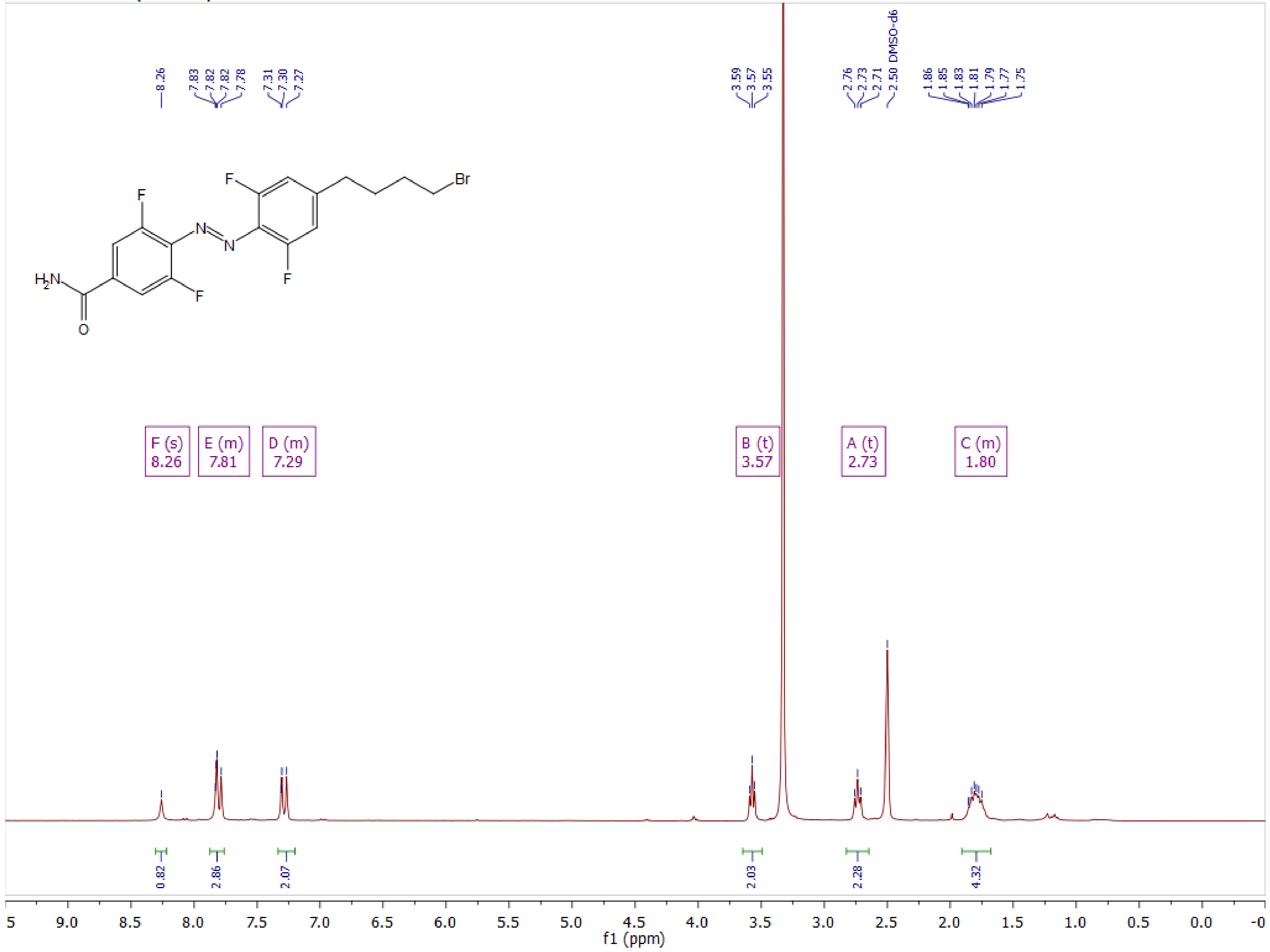

Carbon NMR of 14 (DMSO-d6)

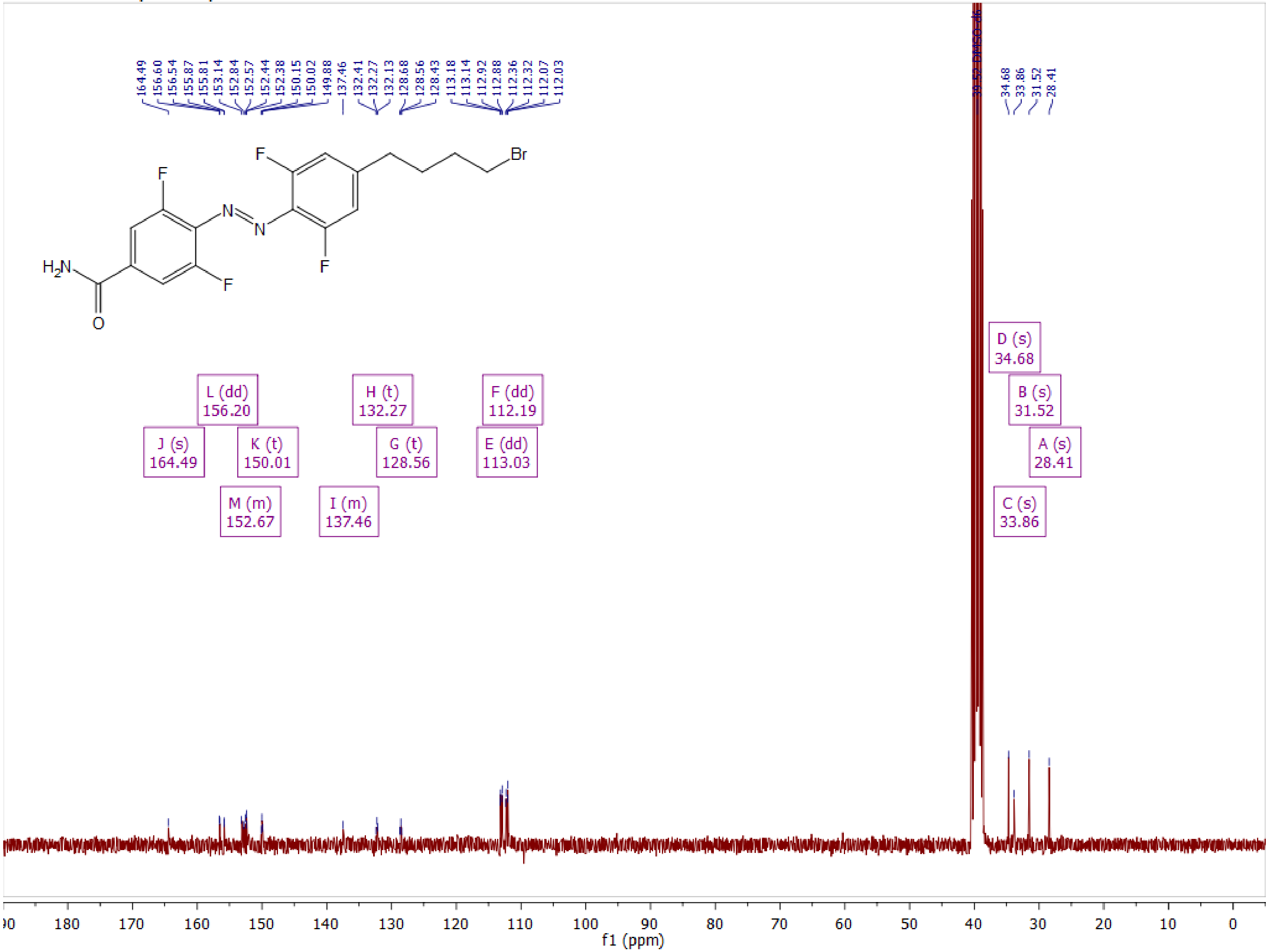

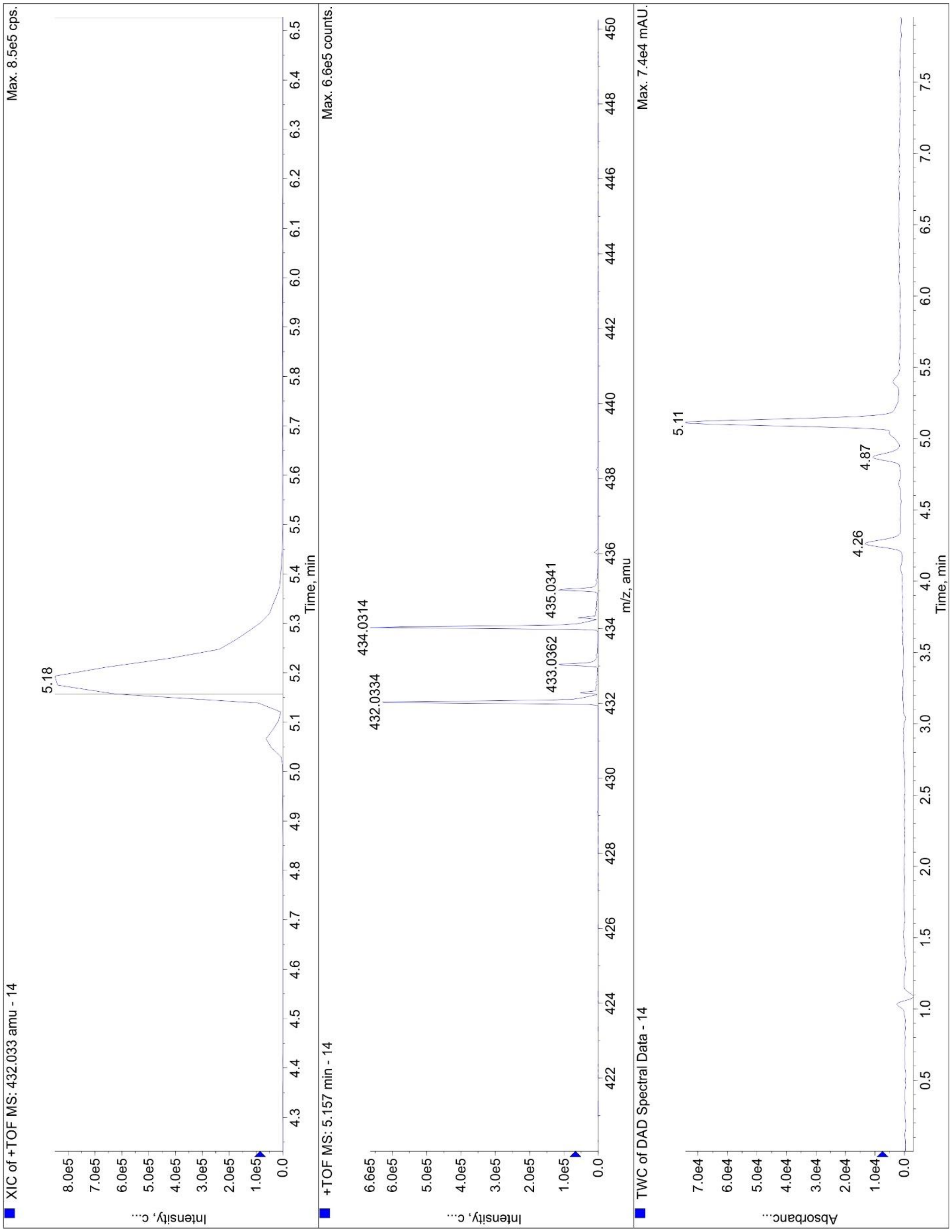
XIC of +TOF MS: 432.033 amu - 14
Max. 8.5e5 cps.
5.18
Intensity, c...
Time, min
+TOF MS: 5.157 min - 14
Max. 6.6e5 counts.
432.0334
433.0362
434.0314
435.0341
Intensity, c...
m/z, amu
TWC of DAD Spectral Data - 14
Max. 7.4e4 mAU.
5.11
4.87
4.26
Absorbanc...
Time, min

Proton NMR of 15 (CD3OD)

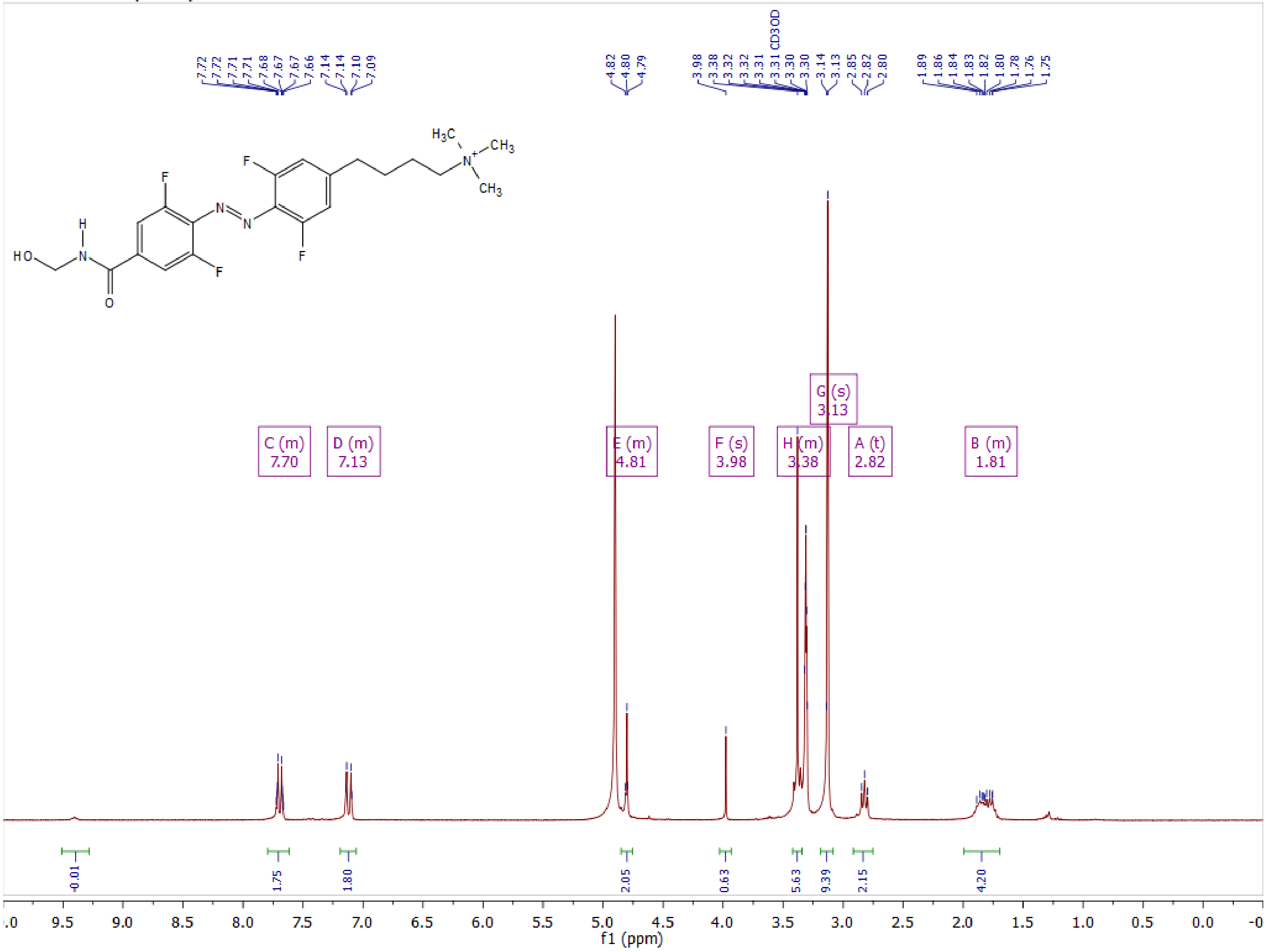

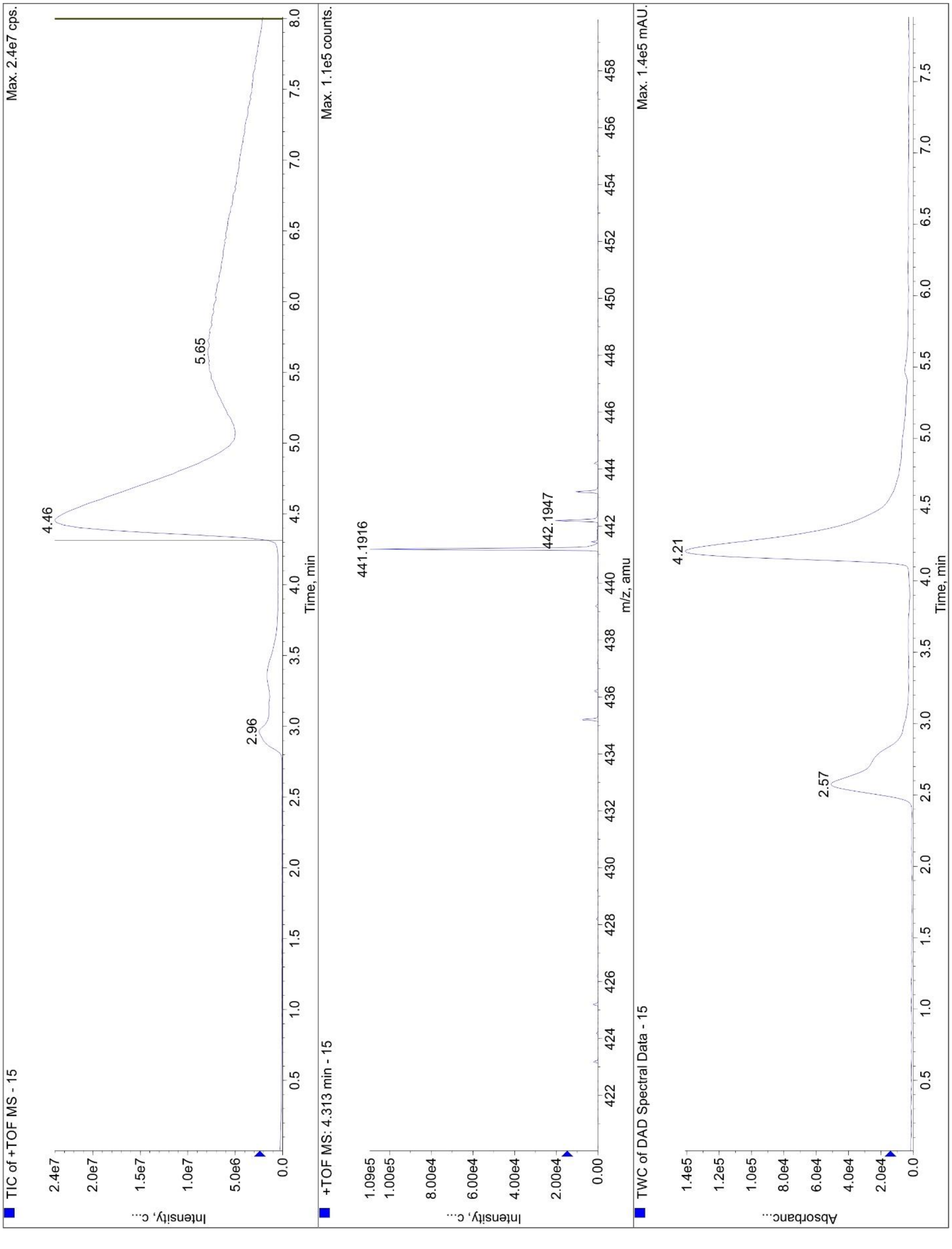
TIC of +TOF MS - 15
Max. 2.4e7 cps.
Intensity, c...
Time, min
4.46
5.65
2.96
+TOF MS: 4.313 min - 15
Max. 1.1e5 counts.
441.1916
442.1947
m/z, amu
TWC of DAD Spectral Data - 15
Max. 1.4e5 mAU.
Absorbanc...
4.21
2.57

References

1 J. Moreno, M. Gerecke, L. Grubert, S. A. Kovalenko, S. Hecht; "Sensitized Two-NIR-Photon Z→E Isomerization of a Visible-Light-Addressable Bistable Azobenzene Derivative" *Angew. Chem., Int. Ed.* **2016**, 55, 1544-1547.

2 K. Negoro, Y. Yonetoku, H. Misawa-Mukai, W. Hamaguchi, T. Maruyama, S. Yoshida, M. Takeuchi, M. Ohta; "Discovery and biological evaluation of novel 4-amino-2-phenylpyrimidinederivatives as potent and orally active GPR119 agonists" *Bioorg. Med. Chem.* **2012**, 20, 5235–5246.
